# Cascade Biorefinery of *Chlorella vulgaris*: Optimized Extraction Sequencing for Sustainable Whole-Biomass Valorization

**DOI:** 10.1021/acssuschemeng.5c13850

**Published:** 2026-03-25

**Authors:** Francesco Del Prete, Francesca Sansone, Francesca Fortunato, Tiziana Esposito, Teresa Mencherini, Annamaria Di Serio, Domenico Ronga, Rita. P. Aquino

**Affiliations:** Department of Pharmacy, University of Salerno, 84084 Fisciano, SA, Italy

**Keywords:** microalgal processing, polarity-driven fractionation, process sequencing optimization, multiproduct recovery, high-value biomolecules, residual biomass retention

## Abstract

An integrated biorefinery model was developed and experimentally validated for the multiproduct valorization of *Chlorella vulgaris* biomass through the sequential and polarity-driven extraction of high-value bioactive fractions. The extraction of *C. vulgaris* biomass was based on integration of mechanical disruption with selective aqueous, ethanol-based, and alkaline extraction steps. Six different extraction sequences were applied to high-quality biomass cultivated in an indoor photobioreactor system to obtain polysaccharides (Po), pigments (Pi), and proteins (Pr). The resulting fractions were compared in terms of extraction yields, biochemical marker content, and postextraction residual biomass also characterized in term of morphology and solid state. The novelty of the work lies in a systematic comparison of all six possible extraction permutations within the polarity-driven cascade. Our results demonstrate that, among all configurations, the Po → Pi → Pr sequence provided the most balanced outcome, yielding 20.7% polysaccharides, 16.2% pigments, and 27.6% proteins. At the same time, 36% (w/w) of residual biomass was preserved, a significantly higher retention compared to the most existing strategies relying on lipid-first or fixed-sequence extraction schemes. The considerable residual biomass (36% w/w), retained after processing remains available for potential further valorization, including lipid extraction or agronomic reuse as a biostimulant. Biochemical integrity was maintained across all fractions, as confirmed by reference markers: 411.07 mg g^–1^ total carbohydrates (Dubois method), 392.53 mg g^–1^ proteins (Bradford assay), and 0.88 mg g^–1^ lutein (UV–Vis). Overall, the findings indicate that a rationally designed cascade biorefinery can maximize product recovery, minimize material losses, and preserve extract quality.

## Graphical abstract

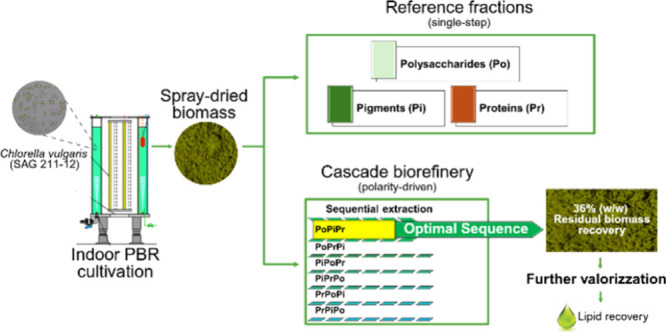

## 1 Introduction

Microalgae such as *Chlorella vulgaris* are photosynthetic microorganisms capable of synthesizing a wide array of bioactive compounds, including polysaccharides, proteins, pigments, and lipids, with expanding applications in the nutraceutical, cosmetic, pharmaceutical, and agronomic sectors.^1–4^ However, large-scale exploitation of *C. vulgaris* remains economically constrained due to the high costs of cultivation and downstream processing, particularly harvesting and lipid extraction.^5,6^ Early microalgal research primarily focused on the extraction of single high-value metabolites, most commonly lipids for biofuel production. However, single-product extractions are rarely cost-effective as confirmed by multiple techno-economic assessments.^7^ Consequently, attention has shifted toward integrated biorefinery models that enable multiproduct recovery from a single biomass source. These approaches aim to improve the economic and environmental viability of microalgal production systems and to enhance the Energy Return On Investment (EROI). They also contribute to reducing waste generation and promoting alignment with circular economy principles.^8–11^ *C. vulgaris* is particularly well suited for cascade processes because it accumulates structurally diverse metabolites with high commercial relevance. These include lipids convertible into biodiesel or sustainable aviation fuels; polysaccharides usable as biostimulants, biopolymer precursors, or bioethanol substrates; and proteins regarded as sustainable alternatives to conventional feed and food proteins.^1^ In addition, the species produces pigments such as lutein for nutraceutical and cosmeceutical applications.^2^ Sequential extraction technologies, typically employing aqueous, ethanol-based, and alkaline treatments, allow selective and mild recovery of these fractions.^12–15^ Even after metabolite extraction, microalgal residues remain rich in structural polysaccharides, minerals, and glycoproteins; they can therefore be valorized as biofertilizers or soil improvers, highlighting the potential to move toward low-waste or near-zero-waste microalgal biorefinery schemes.^4,16^ Several studies have demonstrated that either microalgal extracts and postextraction residuals can enhance seedling development, nutrient uptake, and physiological performance in horticultural species, especially lettuce (*Lactuca sativa* L.), a widely used model due to its rapid growth and sensitivity to biostimulants^3,17–19^ Cascade and polarity-driven extraction strategies for microalgal biorefineries have been previously proposed. However, most studies focus on a single predefined process configuration or prioritize lipid extraction as the initial step, often within biofuel-oriented designs.^7,20,21^ Therefore, limited attention has been devoted to systematically evaluating how extraction sequence affects overall recovery efficiency, metabolite integrity, and residual biomass preservation across multiple product streams.

For this reason, the key advance of the present study is to perform a six-permutation comparison of a polarity-driven extraction cascade from *C. vulgaris* . By evaluating all possible sequences of aqueous (Po polysaccharides), ethanol-based (Pi pigments), and alkaline (Pr proteins) extraction under identical processing conditions, the study isolates extraction order as an independent process variable. This design enables a quantitative assessment of how the position of each fraction within the cascade (primary, secondary, or tertiary step) affects recovery yield, compositional integrity, and residual biomass retention. To the best of our knowledge, a systematic comparison of all possible extraction sequence permutations has not been previously reported for *C. vulgaris* -based biorefineries. Most existing studies instead examine individual extraction steps or a limited number of cascade configurations.^4,22^ In a recent study, Del Prete et al.^23^ developed an optimized aqueous extraction protocol yielding a polysaccharide-rich extract (CHL-A) from high-quality spray-dried *C. vulgaris* biomass cultivated in controlled indoor photobioreactors. Building upon that foundation, the present work advances toward an integrated cascade biorefinery model aimed at biomass valorization. In contrast to the lipid-first sequences commonly described in the literature,^20,21^ the present study adopts a polarity-driven extraction framework. Hydrophilic metabolites are recovered first through aqueous extraction, followed by ethanol-extractable pigments and finally protein fractions obtained via alkaline extraction. This design is intended to reduce cross-interference between extraction phases and to preserve metabolite integrity, while maintaining sufficient residual biomass for further valorization (e.g., lipid recovery or agronomic reuse). The novelty of the present work does not derive from the extraction techniques themselves, as they are well established in microalgal processing. Rather, it lies in the integrated assessment of how process sequencing influences metabolite recovery, extract quality, and residual biomass preservation within a single analytical framework. The sequential framework was optimized according to three key parameters: process efficiency (extraction yields), biochemical fidelity of the recovered metabolites, and postextraction residual biomass. Each extracted class of metabolites was characterized by the determination of the reference marker content (Dubois method for carbohydrates, Bradford assay for proteins, and UV–Vis for lutein) to assess both yield and compositional integrity across the six tested sequences. Furthermore, to evaluate the suitability as secondary coproducts within a circular valorization pathway, such residual biomasses from extraction steps, were investigated for the agronomic potential through biostimulation assays on *Lactuca sativa* L. Overall, this study proposes an integrated biorefinery model for *C. vulgaris* based on an optimized extraction sequence. The approach verifies the spectrum of efficiently recoverable microalgal constituents and assesses the potential for residual biomass reuse.

## 2 Materials and Methods

### 2.1 Materials

All solvents, reagents, and salts were of analytical grade and used as received without further purification. Distilled water and ethanol (EtOH, 96% v/v) were used as extraction solvents. The following reagents were employed in the analytical determinations: phenol reagent, sulfuric acid (H_2_SO_4_, 98%), Bovine Serum Albumin (BSA, bioreagent), Bradford reagent (0.1–1.4 mg mL^–1^ protein), d-(+)-glucose (≥99.5%), lutein analytical standard (purity >96%), sodium hydroxide (NaOH, 2 N), and hydrochloric acid (HCl, 0.1 M).The salts used for culture medium preparation included ammonium nitrate (NH_4_NO_3_), sodium nitrate (NaNO_3_), potassium phosphate dibasic trihydrate (K_2_HPO_4_·3H_2_O), magnesium sulfate heptahydrate (MgSO_4_·7H_2_O), calcium chloride dihydrate (CaCl_2_·2H_2_O), citric acid (C_6_H_8_O7), ferric ammonium citrate (C_6_H_8_FeNO7), ethylenediaminetetraacetic acid (EDTA), sodium carbonate (Na_2_CO_3_), boric acid (H_3_BO_3_), manganese sulfate monohydrate (MnSO_4_·H_2_O), zinc sulfate heptahydrate (ZnSO_4_·7H_2_O), cupric sulfate pentahydrate (CuSO_4_·5H_2_O), and ammonium heptamolybdate tetrahydrate ((NH_4_)_6_Mo7O_2__4_·4H_2_O). All chemicals were purchased from Sigma-Aldrich (Darmstadt, Germany). The *Chlorella vulgaris* biomass used in this study was produced and stabilized at the laboratories of the Department of Pharmacy (Difarma), University of Salerno (Italy). *Lactuca sativa* L. seeds (genotype “Eden”) were obtained from Maraldi Srl (Cesena, Italy). The commercial biostimulant Agrialgae and the unfertilized peat substrate Mannaflor Tray 80/20 0.4 were used in greenhouse experiments.

### 2.2 Microalgal Strain, Cultivation, Harvesting, and Biomass Drying

*C. vulgaris* (strain SAG 211–12, provided by the Culture Collection of Algae at the University of Göttingen, Germany) was cultivated, harvested, and stabilized at the Department of Pharmacy, University of Salerno (Italy). A modified BG11 medium^24^ was used for algal growth. The inoculum was prepared in batch mode using Erlenmeyer flasks with volumes progressively increasing from 100 mL to 2 L, incubated in a controlled climatic chamber (Angelantoni Life Science, model EKOCH 1500, Massa Martana (PG), Italia) at 28 °C, 50% relative humidity, and under continuous artificial illumination (85 μmol m^–2^ s^–1^, 16:8 light/dark cycle). The culture was then scaled up in an experimental photobioreactor system consisting of an “acceleration module” (three vertical column reactors of 8, 16, and 32 L operated in batch mode) and a “production module” (three 150 L annular photobioreactors operated semicontinuously for 28–30 days). Continuous air insufflation (2–5 L min^–1^) was provided using a linear membrane compressor (Air Pump model DBP40 Air Mac, Changhua, Taiwan) to maintain suspension and aeration. Cell growth was monitored daily by cell counting (Counting chamber Bürker, Fisher scientific, Milano, Italia), optical density (560 and 750 nm), and dry weight (filtration on 0.45 μm filters and drying at 80 °C). At the stationary phase, biomass was harvested by continuous centrifugation (Extreme Algae Centrifuge 230 V, Algae Centrifuge, Sacramento, CA, USA) with separation efficiencies exceeding 90%. The spent culture medium was recycled after nutrient replenishment to enhance sustainability and reduce production costs. The collected algal paste (∼80% moisture) was dried using a Büchi B-290 mini Spray Dryer (Büchi Laboratoriums-Technik, Flawil, Switzerland) following optimized parameters.^23,25^ The process achieved yields above 75% (w/w) of dried microalgal biomass. The resulting fine powder was stabilized in a humidity- and temperature-controlled chamber for 72 h until reaching a residual moisture content below 3%. This standardized procedure produced a stable, homogeneous biomass suitable for subsequent sequential extraction. The high quality and shelf stability of the dried biomass ensured reproducibility in downstream extraction and analytical steps.

### 2.3 Extract Preparation

Extraction processes were designed to recover three biologically relevant fractions, polysaccharides, pigments, and proteins, from the spray-dried biomass of *C. vulgaris* , targeting both intracellular and cell wall components.

#### 2.3.1 Aqueous Extraction (Polysaccharides) of *C. vulgaris* Biomass

The polysaccharide-rich extract (CHL-A) was prepared following the optimized protocol previously described by Del Prete et al.^23^ Briefly, spray-dried *C. vulgaris* biomass was subjected to combined mechanical disruption using Ultraturrax homogenization and vortexing with quartz beads, followed by hot-water extraction at 80 °C for 2 h under constant agitation. The aqueous supernatants obtained from three consecutive extraction cycles were combined, concentrated under reduced pressure, filtered (0.45 μm), precipitated with four volumes of ethanol, and stored at 4 °C for 12 h. The resulting precipitate was centrifuged (10,000 rpm, 30 min), resuspended in distilled water, and freeze-dried to obtain the CHL-A extract. The method ensured efficient recovery of neutral water-soluble polysaccharides while preserving their structural integrity. After each aqueous extraction, the remaining solid biomass was recovered, freeze-dried, and subsequently used in the following extraction steps or for agronomic evaluation as a potential natural biostimulant.

##### 2.3.1.1 Determination of Reference Marker Content (Total Neutral Carbohydrates)

The neutral carbohydrate content of CHL-A was determined using the phenol–sulfuric acid colorimetric method as described by Del Prete et al.,^23^ employing d-(+)-glucose as the reference standard. Results were expressed as mg glucose equivalents per gram of dry extract.

#### 2.3.2 Ethanol-Based Extraction (Pigments) of *C. vulgaris* Biomass

Pigments were extracted using an ethanol-based protocol combined with mechanical and acoustic cell disruption. A total of 0.5 g of spray-dried *C. vulgaris* biomass was suspended in 25 mL of 96% ethanol in a 50 mL Falcon tube. The suspension was homogenized using an Ultraturrax (T25 IKA, Staufen, Germany) for 60 s at 17000 rpm and vortexed for 5 min with 10 g of 1 mm quartz beads. An additional 25 mL of ethanol was added, reaching a final biomass-to-solvent ratio of 1:100 in an amber flask. The mixture was subjected to sonication for 15 min, followed by magnetic stirring at 350 rpm for 30 min on a heated plate (IKA RCT Standard, Sigma-Aldrich, Germany). After each extraction cycle, samples were centrifuged (FRONTIER 5916R, OHAUS, Nänikon, Switzerland) at 5000 rpm for 10 min at 4 °C. The pellet was extracted twice as much following the same procedure. Supernatants were combined, filtered (0.45 μm PTFE filters, Sartorius), and evaporated to dryness at 30 °C using a rotary evaporator (Hei-VAP, Heidolph, Schwabach, Germany). The resulting extract was weighed to determine yield and used for pigment quantification. After each ethanol-based extraction, the residual biomass was collected, freeze-dried, and preserved for the subsequent extraction stage or for agronomic evaluation of its potential biostimulant activity.

##### 2.3.2.1 Determination of Reference Marker Content (Lutein)

The ethanol-based pigment-rich extract (CHL-P) was characterized by UV–vis spectrophotometry to determine its lutein content, used as a biochemical reference marker. Absorbance spectra were recorded between 200 and 700 nm using ethanol as solvent (stock solution: 1 mg mL^–1^) and a Specord 200 Plus UV/vis spectrophotometer (Sotax, Analytik Jena, Germany). Lutein (analytical standard, purity >96%, Sigma-Aldrich) was used to prepare the calibration curve in ethanol, with concentrations ranging from 75 to 500 mg L^–1^. Absorbance readings were taken at λ = 423 nm using 1 mm quartz cuvettes (Hellma Analytics, Milano, Italia).

Lutein concentration in the extracts was determined according to the Beer–Lambert law (*C* = *A/*ε*l*), where *A* is the absorbance, ε the molar extinction coefficient, and *l* the optical path length. The calibration curve showed a strong linear correlation between concentration and absorbance, described by the regression equation:

$$A=1.04\times 10^{-3}C+0.034\;(R^{2}=0.994)$$

This high coefficient of determination confirmed the excellent linearity and reliability of the method for quantitative analysis of lutein in the ethanol-based extracts.

#### 2.3.3 Alkaline Extraction (Proteins) of *C. vulgaris* Biomass

A protein-rich fraction was extracted from dried *C. vulgaris* biomass using an alkaline protocol. A total of 0.5 g of biomass powder was suspended in 10 mL of 2N NaOH (pH 12) and homogenized using an Ultraturrax T25 (small rotor) for 60 s at 17000 rpm. The rotor was rinsed with an additional 10 mL of NaOH, collected in the same Falcon tube. A further 5 mL of NaOH and 10 g of 1 mm quartz beads were added. The mixture was vortexed (LBX V05, Labbox Labware, Barcelona, Spain) for 5 min and incubated at 40 °C on a heated plate (IKA RCT Standard, Sigma-Aldrich, Germany) at 350 rpm for 2 h.

After incubation, excluding the beads, the sample was centrifuged (FRONTIER 5916R, OHAUS, Nänikon Switzerland) at 6000 rpm for 20 min. The pellet was extracted two additional times under identical conditions. The pooled supernatants, free from quartz beads, were centrifuged again at 6000 rpm for 20 min and acidified to pH 3 with 0.1 M HCl to induce protein precipitation. After centrifugation (30 min, 6000 rpm), the resulting pellet was neutralized to pH 7 using double-distilled water and 0.1 M NaOH. The final extract (CHL-Alk) was frozen and freeze-dried (Buchi Lyovapor L-200, Switzerland). Protein yield was expressed as the percentage weight of extract relative to initial dry biomass. After the alkaline extraction, the remaining solid fraction was recovered, washed, freeze-dried, and stored for potential reuse in other sequential extraction schemes.

##### 2.3.3.1 Determination of Reference Marker Content (Total Proteins)

The CHL-Alk extract was analyzed for total protein content using the Bradford colorimetric assay.^26^ This method is based on the interaction between Coomassie Brilliant Blue G-250 dye and specific amino acid residues, resulting in a shift in absorbance from 465 nm (cationic form) to 595 nm (anionic protein-bound form), which is directly proportional to protein concentration. The extract was diluted to 1 mg mL^–1^ in double-distilled water, and appropriate aliquots were combined with 500 μL of Bradford reagent and distilled water to a final volume of 1 mL. Absorbance was measured at 595 nm using a UV–vis spectrophotometer (Evolution 201, Thermo Fisher Scientific, Milano, Italy) with 10 mm quartz cuvettes. Bovine Serum Albumin (BSA) was used as the standard protein in the concentration range of 2–12 μg mL^–1^ to construct the calibration curve. A strong linear relationship was observed between absorbance and protein concentration, expressed by the regression equation:

$$A=0.0524C+0.0886\;(R^{2}=0.9975)$$

Protein concentrations in the extracts were calculated by interpolation from this calibration equation. The high correlation coefficient confirmed the accuracy and reliability of the method for quantifying total proteins in the CHL-Alk extracts.

### 2.4 Sequential Extractions of *C. vulgaris* Biomass

Six different extraction sequences were organized into three logical pairs (Figure 1), in which each pair begins by extracting a different biologically relevant fraction:

- PoPiPr and PoPrPi: beginning with polysaccharides
- PiPoPr and PiPrPo: beginning with pigments
- PrPoPi and PrPiPo: beginning with proteins

**Figure 1:**
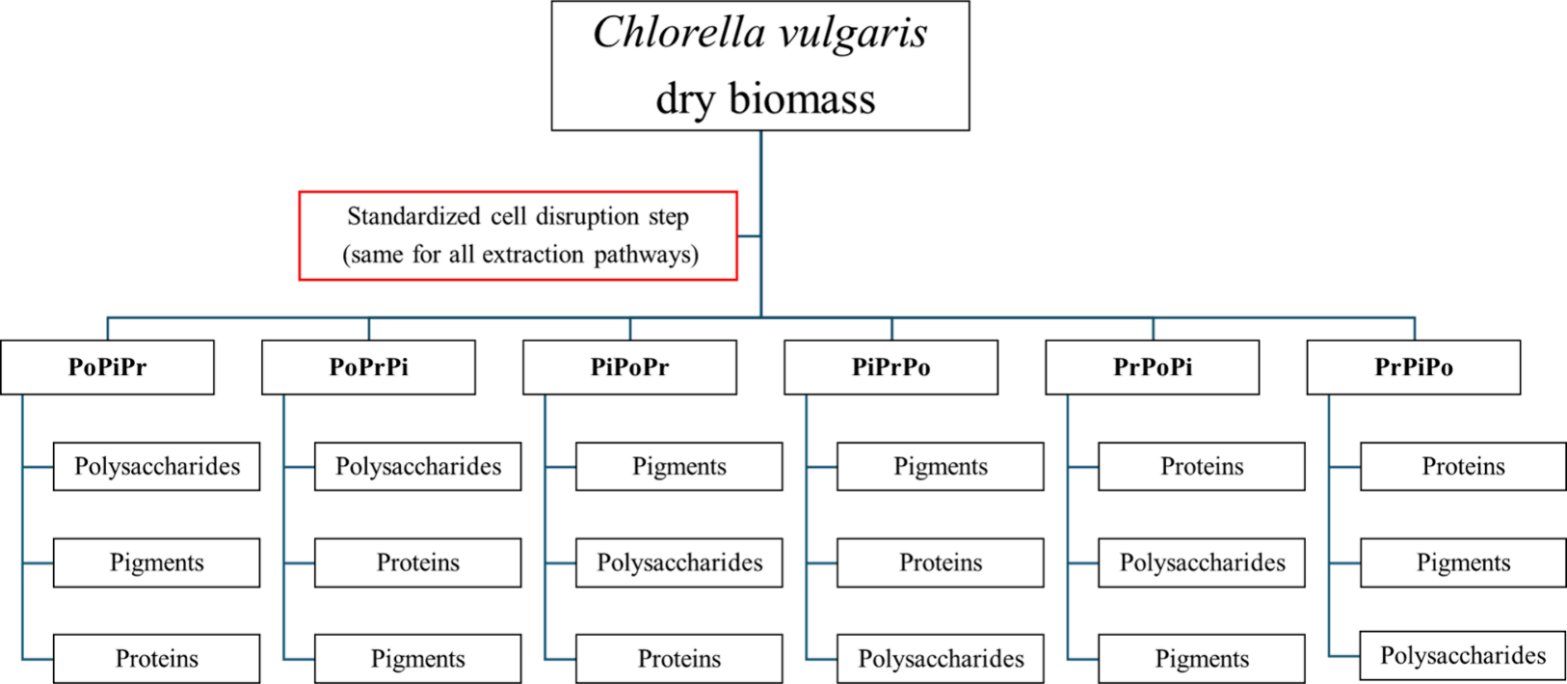
Extraction sequence design for stepwise recovery of polysaccharides (Po), pigments (Pi), and proteins (Pr) from **Chlorella vulgaris*.*

Each extraction step was carried out according to the protocols detailed in Sections 2.3.1–2.3.3. Before each individual extraction step, a standardized cell disruption procedure was applied to ensure consistent accessibility of the biomass and to maximize extraction efficiency across all sequences. After each extraction, the residual biomass was collected by centrifugation (FRONTIER 5916R, OHAUS, Nänikon Switzerland, 6000 rpm, 10 min), freeze-dried (Buchi Lyovapor L-200, Flawil, Switzerland), and either used for the next extraction or stored for agronomic evaluation.

### 2.5 Morphological Analysis of Biomass—Scanning Electron Microscopy (SEM)

The morphology of the dried *C. vulgaris* biomass before and after the sequential extraction processes was evaluated by SEM. Samples were observed using a Carl Zeiss EVO MA 10 microscope equipped with a secondary electron detector (Carl Zeiss SMT Ltd., Cambridge, UK). Prior to imaging, samples were coated with a thin layer of gold (200–400 Å) using an EMSCD005 sputter coater (Leica Microsystems, Milano, Italy). Imaging was performed under high vacuum at an accelerating voltage of 20 keV.

### 2.6 Thermal Analysis—Differential Scanning Calorimetry (DSC)

Differential scanning calorimetry (DSC) analyses were performed to investigate the thermal behavior and moisture-related transitions of residual biomasses obtained after aqueous, ethanolic, and alkaline extraction. Approximately 2 mg of each sample were weighed using a MTS Mettler Toledo microbalance (Worthington, OH, USA) and placed into hermetically sealed aluminum pans. Measurements were carried out using an indium-calibrated Mettler Toledo DSC 822e instrument (Mettler Toledo, OH, USA) under a nitrogen atmosphere. Samples were first subjected to a dynamic heating scan from 25 to 350 °C at a rate of 10 °C min^–1^ to evaluate thermal transitions and moisture-related endothermic events. To distinguish intrinsic matrix transitions from water evaporation effects, a controlled dehydration step was applied. Samples were heated from 30 to 100 °C at 10 °C min^–1^ and held at 100 °C for 5 min under nitrogen flow. After dehydration, a second dynamic heating scan was performed under the same conditions. An empty sealed pan was used as reference. Thermal transitions were analyzed using the instrument software (STARe software for DSC 822e, Mettler Toledo).

### 2.7 Evaluation of Phytotoxicity and Agronomic Response of Residual Biomasses

A preliminary phytotoxicity assay was performed according to Zucconi^27^ to assess the safety of the residual *C. vulgaris* biomasses recovered at each extraction stage before their agronomic application. Aqueous samples were prepared by dissolving 2.5 mg of microalgal powder in 50 mL of distilled, sterilized water, sonicated for 30 min (Grant Ultrasonic Bath XUBA3, Dominique Dutschher, Bruxelles, France), and centrifuged at 2000 rpm for 5 min (IEC CL30R, Thermo Scientific, Milano, Italy). The supernatant was collected, and serial dilutions (1:10, 1:100, 1:1000, v/v) were prepared in distilled water. Fifteen *Lactuca sativa* L. seeds (cv. Eden, Maraldi Srl, Italy) were placed in Petri dishes lined with filter paper moistened with 4 mL of each extract, while distilled water served as the control. Dishes were incubated in darkness at 27 °C for 36 h, and germination rate and root length were measured with a digital caliper. The Germination Index (GI%) was calculated following.^27^

Based on these results, the highest nonphytotoxic dilution (1:1000) was selected for greenhouse testing. The experiment was carried out under controlled conditions (25–28 °C, 37–50% relative humidity, 16 h light/8 h dark photoperiod) using a mobile meteorological station (PCE-FWS 20N, PCE Instruments, Hirschau, Germany). *Lactuca sativa* L. (cv. Eden) seeds were sown in 500 mL pots (10 cm diameter) containing unfertilized peat substrate (Mannaflor Tray 80/20 0.4). Each treatment included five biological replicates. Irrigation was performed three times per week with 125 mL of water (pH 7.34; EC 518,4 μS/cm), and fertilization was applied twice with 0.13 g N (as NH_4_NO_3_) at sowing and after 10 days.

Foliar applications were carried out weekly from the appearance of the first true leaf until harvest (28 days after germination). Treatments were as follows:

T1- Control 1 (distilled water);
T2 - Control 2 (commercial biostimulant Agrialgae);
T3 - CHL-TQ (1:1000);
T4 - CHL-RA (1:1000);
T5 - CHL-RET (1:1000).

Where CHL-TQ = whole *C. vulgaris* biomass; CHL-RA = residual *C. vulgaris* biomass after aqueous extraction, and CHL-RET = residual *C. vulgaris* biomass after ethanol-based extraction.

The commercial biostimulant Agrialgae was applied at the manufacturer-recommended dilution for foliar application (1:1000), ensuring agronomically relevant and comparable operating conditions.

At harvest, agronomic and physiological parameters were recorded. The number of leaves per plant, plant height, and fresh weight of shoots (aboveground, g pot^–1^) and roots (belowground, g pot^–1^) were measured. Soil moisture was determined using a FieldScout TDR sensor (Spectrum Technologies, USA). Stomatal conductance and chlorophyll index were measured at the leaf level using a Delta-T AP4 porometer (Delta-T Devices, UK) and a SPAD-502 m (Konica Minolta, Japan), respectively. Crop water productivity (CWP) and nitrogen agronomic efficiency (NAE) were calculated according to Ronga et al.^28^

### 2.8 Statistical Analysis

Data are presented as mean ± standard deviation (SD) of three independent replicates (*n* = 3). Before statistical analysis, the Normality of residuals was assessed using the Shapiro–Wilk and QQ plot tests, and the homogeneity of variances was evaluated using the Brown–Forsythe test. Differences among groups were analyzed using ordinary one-way ANOVA followed by Tukey’s HSD post hoc test. Differences were considered statistically significant at *p* < 0.05. Statistical analyses were performed using GraphPad Prism 10.0 (GraphPad Software, San Diego, CA, USA).

## 3 Results and Discussion

### 3.1 Production and Stabilization of *Chlorella vulgaris* Biomass

*C. vulgaris* was cultivated in an indoor system of integrated photobioreactors under controlled temperature, light, and aeration conditions, as previously described by Del Prete et al.^23^ Cultures were maintained for approximately 28 days, progressing through batch and semicontinuous growth phases until reaching the early stationary stage. At the end of each cultivation cycle, biomass was harvested via continuous centrifugation, yielding approximately 4 g L^–1^ of wet biomass. The resulting biomass paste (∼80% moisture) was then spray-dried, producing a fine, shelf-stable powder with a ∼20% dry-mass yield and residual moisture below 4%. The spray-drying process ensured high product stability and preserved the physicochemical integrity necessary for downstream extraction. This high-quality dry biomass served as the substrate for the sequential extraction processes developed in this study.

### 3.2 Individual Extraction

The stable spray-dried *C. vulgaris* biomass was first subjected to three individual extraction procedures, aqueous, ethanol-based, and alkaline, to establish the maximum recoverable yields and respective biochemical profiles of the polysaccharides, pigments, and proteins rich fractions. These values served as the reference baseline for evaluating the performance of the sequential extraction schemes presented in the following sections.

#### 3.2.1 Aqueous Extraction (Polysaccharides)

Under the previously optimized conditions (Section 2.3.1; Del Prete et al.^23^), the polysaccharide-rich extract CHL-A was obtained with a yield of 20.72 ± 0.66% (w/w) and a neutral carbohydrate content of 411.07 ± 7.79 mg glucose eq g^–1^. These values confirm both the efficiency of the extraction protocol and the high abundance of water-soluble polysaccharides in the spray-dried biomass. To support the extraction results, scanning electron microscopy (SEM) was employed to assess the morphological evolution of *C. vulgaris* before and after the mechanical disruption step. This operation represents a core unit process in all subsequent extraction procedures. As shown in Figure 2, the spray-dried biomass (Figure 2A) retained its characteristic spheroidal morphology. Despite the presence of typical dehydration-induced features, surface wrinkling, collapse, and corrugation, cellular contours remained intact, indicating that spray drying does not mechanically rupture the microalgal cell wall. This preservation is consistent with reports describing spray-dried *Chlorella* powders as structurally compact and mechanically resistant, owing to their polysaccharide and glycoprotein rich envelopes. Conversely, the biomass subjected to mechanical pretreatment (Figure 2B) displayed severe structural disruption, with ruptured, fragmented, and collapsed cells. The emergence of larger cluster aggregates suggests postlysis phenomena such as agglomeration, crystallization, or precipitation of intracellular and extracellular compounds. These phenomena could have been promoted by multistep aqueous processing that facilitates the reorganization of solubilized macromolecules or minerals into larger assemblies. This extensive structural damage confirms the effectiveness of the mechanical disruption strategy employed. The approach combines shear forces generated by Ultraturrax homogenization with the percussive impact of quartz beads in aqueous suspension. The resulting high-energy collisions physically disrupt the rigid microalgal cell wall. This structural breakdown enhances solvent penetration and facilitates the release of intracellular metabolites, including polysaccharides, pigments, and proteins. Importantly, this mechanical approach avoids harsh chemicals and high temperatures, thereby protecting thermolabile compounds.

**Figure 2:**
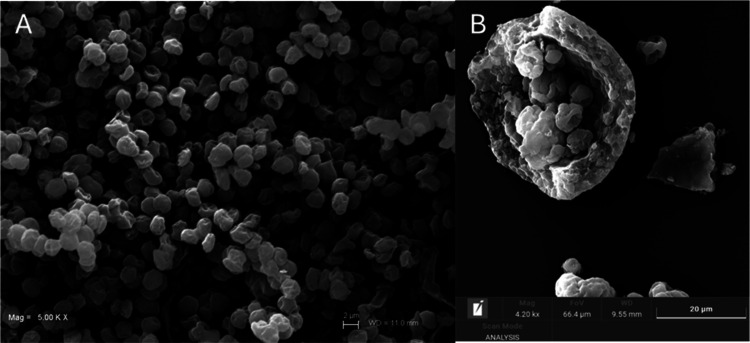
SEM images of *Chlorella vulgaris* biomass: (A) spray-dried powder showing intact but surface-collapsed cells and (B) mechanically disrupted biomass displaying ruptured and fragmented cell walls.

SEM analysis confirmed that spray drying preserves cellular integrity, whereas mechanical disruption effectively compromises the cell wall, enabling high extraction yields. Following mechanical disruption and aqueous processing, the CHL-A extract was designated as the primary fraction (Po) within the sequential biorefinery workflow. This fraction served both as a reference and as the starting point for evaluating how subsequent extraction steps and their corresponding residual biomasses contributed to overall cascade valorization efficiency.

#### 3.2.2 Ethanol-Based Extraction (Pigments)

Under the optimized conditions described in Section 2.3.2, ethanol-based extraction produced a pigment-rich fraction (CHL-P) with a yield of 18.36 ± 0.27% (w/w). UV–Vis analysis confirmed a high carotenoid content. Lutein was quantified at 1.09 ± 0.05 mg g^–1^, consistent with values typically reported for *C. vulgaris* . These results indicate that ethanol efficiently solubilized intracellular pigments and ensured their effective recovery. CHL-P extract was used as the pigment reference fraction in the sequential biorefinery and generated the corresponding residual biomass for the subsequent extraction step.

#### 3.2.3 Alkaline Extraction (Proteins)

Optimization of the alkaline extraction conditions (Section 2.3.3) resulted in recovery of the CHLA fraction with a yield of 40.16 ± 0.83% (w/w). The fraction exhibited a total protein content of 360.27 ± 4.36 mg g^–1^. These values are consistent with the typical protein range reported for *Chlorella* biomass (40–60% DW).^4,15^ The strong solubilizing capacity of NaOH facilitated effective protein release from the cell-wall matrix.

However, the alkaline treatment resulted in a residual biomass exhibiting a sticky, highly aggregated consistency and reduced water dispersibility. This physical state made it unsuitable for direct agronomic application or aqueous-based evaluations. Nevertheless, the material remains processable for further downstream treatments involving nonaqueous or harsher extraction conditions (e.g., lipid recovery), provided that appropriate neutralization and conditioning steps are applied.

#### 3.2.4 Physical and Thermal Characterization of Post-Extraction Residual Biomass

To better understand the distinct physical behavior of residual biomasses obtained after each extraction step, a comparative morphological and thermal assessment was performed. Macroscopic imaging (Figure 3) and differential scanning calorimetry (DSC) analyses (Figures 4 and 5 ) were conducted on residues derived from aqueous, ethanol-based, and alkaline treatments. This combined approach enabled correlation of the visible aggregation state and dispersibility of each residue with its thermal transitions and moisture-related behavior. These analyses provided insight into the physical modifications induced by the different extraction conditions.[Fig fig5]

**Figure 3:**
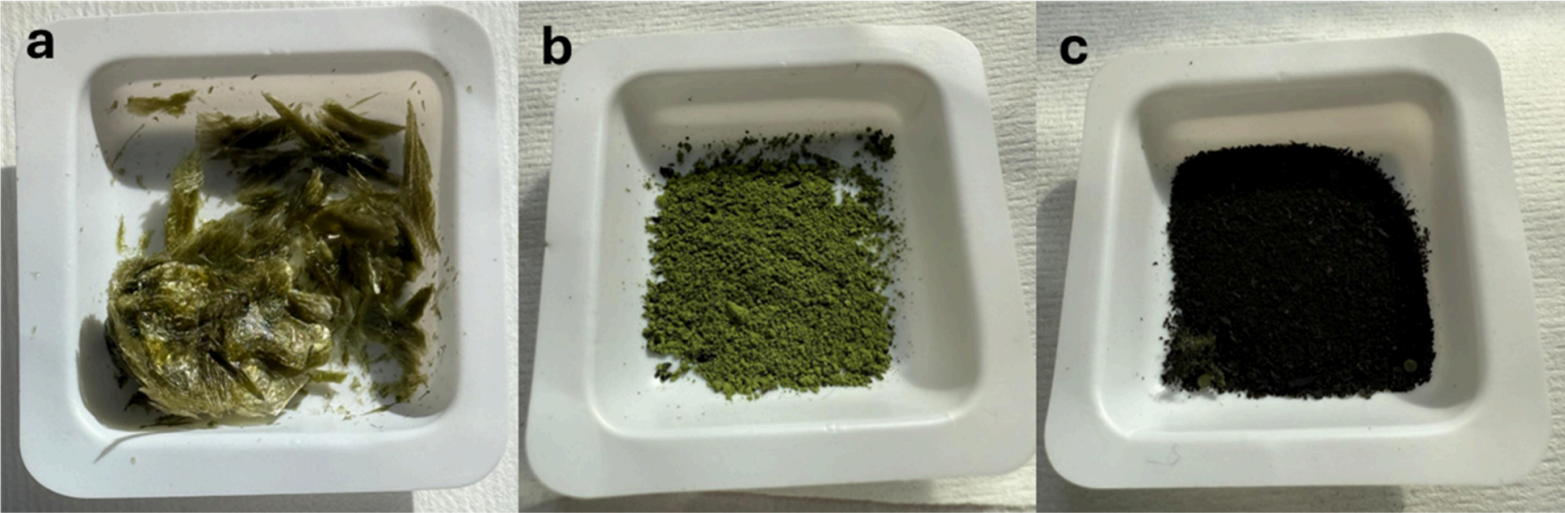
Macroscopic appearance of residual biomasses after (a) alkaline, (b) ethanol-based, and (c) aqueous extraction.

**Figure 4:**
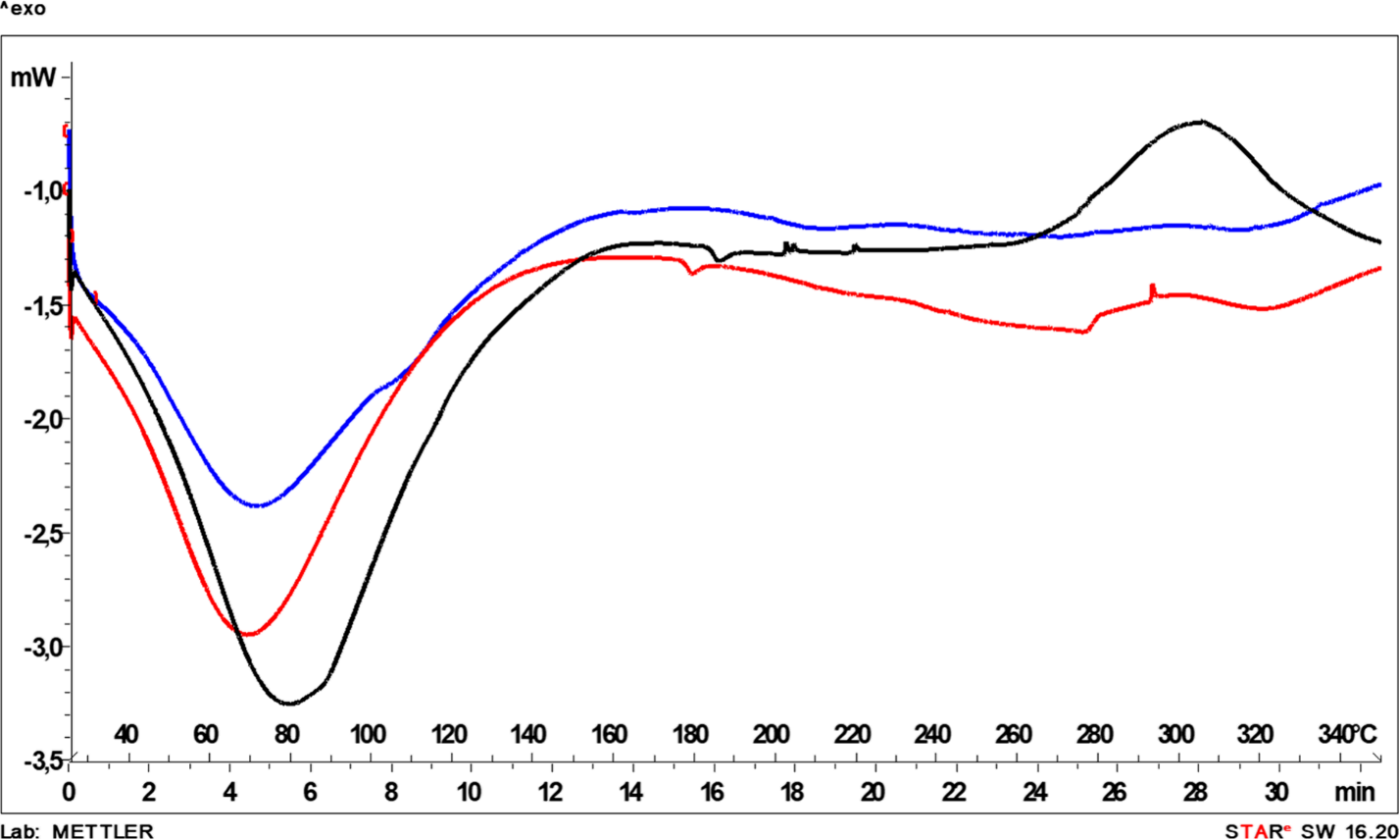
First dynamic DSC scan of residual biomasses (without dehydration cycle) obtained after alkaline (black), ethanol-based (red), and aqueous (blue) extraction.

**Figure 5:**
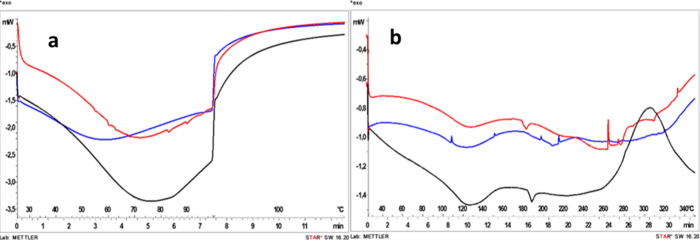
DSC profiles of residual biomasses obtained after alkaline (black), ethanol-based (red), and aqueous (blue) extraction in controlled dehydration (until 100 °C) (a) and subsequent dynamic scan (b).

Figure 3 shows the macroscopic appearance of the residual biomasses obtained after the three extraction steps. The alkaline-treated residue (Figure 3a) exhibits a cohesive and highly aggregated structure, forming compact clusters with evident material self-association. In contrast, the ethanol-derived residue (Figure 3b) appears as a powder with limited particle aggregation. The aqueous residue (Figure 3c) similarly maintains a particulate morphology, although darker in color, and does not display the pronounced clustering observed in the alkaline-treated sample. The marked difference in aggregation state suggests that the extraction medium significantly influences the physical organization of the residual matrix. In particular, the cluster formation observed in the alkaline-treated biomass is consistent with its reduced dispersibility and altered handling behavior, highlighting a distinct physical state compared to the residues obtained after aqueous and ethanol-based treatments. These differences were further investigated by differential scanning calorimetry (DSC) (Figures 4 and 5 ) to correlate the macroscopic aggregation behavior with the thermal transitions and moisture-related properties of each material.[Fig fig5]

Following the initial dynamic scan (Figure 4), all residues exhibited an endothermic event below approximately 100 °C, attributed to moisture evaporation. The alkaline-treated residue (black line) displayed the most intense and broadened endothermic signal. This pattern indicates higher overall moisture retention and a more heterogeneous water distribution compared to the aqueous (blue line) and ethanol-based (red line) residues. To better distinguish intrinsic structural transitions from moisture-related effects, a dehydration cycle (until 100 °C) (Figure 5a) was applied prior to a second dynamic scan (Figure 5b). After controlling dehydration, the thermal profiles of the residues became more clearly differentiated.

Despite the preliminary dehydration step at 100 °C (Figure 5a), the alkaline-treated residue exhibited an additional endothermic contribution in the 120–130 °C range during the subsequent dynamic scan (Figure 5b). This transition is associated with more strongly bound water. In contrast, this contribution was less pronounced in the aqueous and ethanol-based residues (blue and red lines, Figure 5b). The presence of residual bound water suggests enhanced matrix–water interactions in the alkaline-treated biomass. This effect likely arises from structural reorganization and increased exposure of polar functional groups. Alkaline treatment is known to disrupt intermolecular interactions, cleave ester linkages, and alter supramolecular organization. These modifications increase the availability of polar sites capable of forming strong water–matrix interactions.^29^ Such retained bound water may contribute to matrix plasticization and promote aggregation phenomena, thereby explaining the cohesive and sticky physical state observed macroscopically. Importantly, although these results indicate altered physical organization and moisture interactions, they do not necessarily imply reduced chemical stability, which would require dedicated investigation. Accordingly, the CHL–Alk residual biomass was used exclusively as the protein reference fraction within the sequential biorefinery. Its reduced aqueous dispersibility limited direct evaluation in agronomic applications.

### 3.3 Cascade Extraction

An extraction sequence was defined (Figure 1), and the sequential experiments described in Section 2.4 were performed to evaluate the influence of extraction order. Specifically, the analysis focused on recovery yields and the quality of polysaccharide, pigment, and protein fractions obtained from *C. vulgaris* dry biomass. The amount of residual biomass preserved after each step was also assessed. The six extraction sequences were designed to represent all possible permutations of the three target metabolite classes (Po, Pi, Pr). This approach enabled systematic evaluation of extraction position effects independent of solvent type. The design was based on the hypothesis that extraction severity progressively increases from aqueous to ethanol-based and alkaline treatments. Such progression is expected to induce cumulative structural alterations in the microalgal cell wall. Consequently, metabolites recovered in secondary or tertiary positions are expected to exhibit reduced extractability. This effect may result from prior solubilization, depolymerization, or structural collapse of the matrix. Similar cumulative effects of sequential processing have been reported in microalgal and lignocellulosic biorefineries. In these systems, early stage treatments strongly influence downstream recovery efficiency.^2,4,22^ The experimental layout (Figure 1) was designed to enable direct comparisons among sequences starting from the same initial fraction. This structure allowed isolation of the effect of each subsequent extraction step on overall process efficiency.

### 3.4 Effect of Extraction Sequence on Metabolite Yields

Sequential extraction experiments were performed to quantify the influence of extraction order on metabolite recovery from spray-dried *C. vulgaris* biomass. The analysis focused on polysaccharides, pigments, and proteins. Results presented in Figure 6 and Supporting Information (Table S1) indicate that the extraction sequence strongly affects overall recovery efficiency. The highest yields were obtained when each target compound was extracted as the first step of the cascade. Under these conditions, recoveries reached 20.72 ± 0.66% for polysaccharides, 18.36 ± 0.27% for pigments, and 40.16 ± 0.83% for proteins (Figure 7). These values represent the maximum recoveries achievable from untreated biomass. Subsequent extractions led to progressively lower yields, with the extent of loss depending on the extraction order. For polysaccharides, recovery in the secondary stage decreased to 18.38 ± 0.42% after pigment extraction (PiPoPr) and to 13.25 ± 0.65% after protein extraction (PrPoPi), corresponding to reductions of 11 and 36%, respectively. Only the latter showed a statistically significant decrease compared with the primary extraction (*p* < 0.01). This finding suggests that pigment extraction has minimal impact on polysaccharide recovery, whereas protein extraction markedly reduces it. In the tertiary step, polysaccharide yields declined further to 15.23 ± 0.36% (PiPrPo) and 12.06 ± 0.76% (PrPiPo), both significantly lower than the primary yield (*p* < 0.01), with a notable difference between the two sequences (PrPiPo < PiPrPo, *p* < 0.05). Pigment recovery followed a comparable trend. The primary extraction yielded 18.36 ± 0.27%, while secondary extraction after polysaccharides (PoPiPr) resulted in 16.24 ± 0.76% and after proteins (PrPiPo) in 14.82 ± 0.59%, with reductions of 12 and 19%, respectively. Only the latter was statistically different from the primary step (*p* < 0.05). When pigments were extracted last, yields decreased to 14.42 ± 0.73% (PoPrPi) and 13.28 ± 0.87% (PrPoPi), both significantly lower than the first extraction (*p* < 0.01). The decline is likely due to thermal and alkaline stress in earlier steps, promoting oxidation and saponification of carotenoids and chlorophylls.^30–32^ Protein yields displayed a similar behavior, showing a progressive decrease with extraction order. The highest yield (40.16 ± 0.83%) was obtained when proteins were recovered first. Secondary extractions yielded 30.27 ± 0.65% (PoPrPi) and 31.85 ± 0.72% (PiPrPo) (*p* < 0.01), while tertiary extractions decreased further to 27.56 ± 0.34% (PoPiPr) and 20.38 ± 0.64% (PiPoPr) (*p* < 0.001), with significant differences between the two tertiary sequences (*p* < 0.05). These reductions can be attributed to protein denaturation and aggregation caused by prior aqueous and ethanol-based treatments, which alter cell permeability and limit subsequent solubilization.^4,22,33,34^ Overall, the extraction order significantly influenced both metabolite yield and compositional integrity. Alkaline treatments, while effective for protein solubilization, induced irreversible structural alterations in the cell wall that hindered polysaccharide and pigment recovery in later steps. The results align with previous compositional data for **C. vulgaris*,* ^35^ which reported protein contents around 41% and carbohydrate contents between 21 and 29%. Among all tested schemes, PrPiPo (proteins → pigments → polysaccharides) and PrPoPi (proteins → polysaccharides → pigments) provided the highest overall yields, with protein recovery of approximately 40%. However, subsequent pigment and polysaccharide recoveries were substantially reduced. These results confirm that prioritizing protein extraction at the beginning of the cascade maximizes protein yield. At the same time, it compromises the accessibility and solubility of the remaining bioactive fractions. This outcome reinforces the need for careful sequence optimization in microalgal biorefinery design.

**Figure 6:**
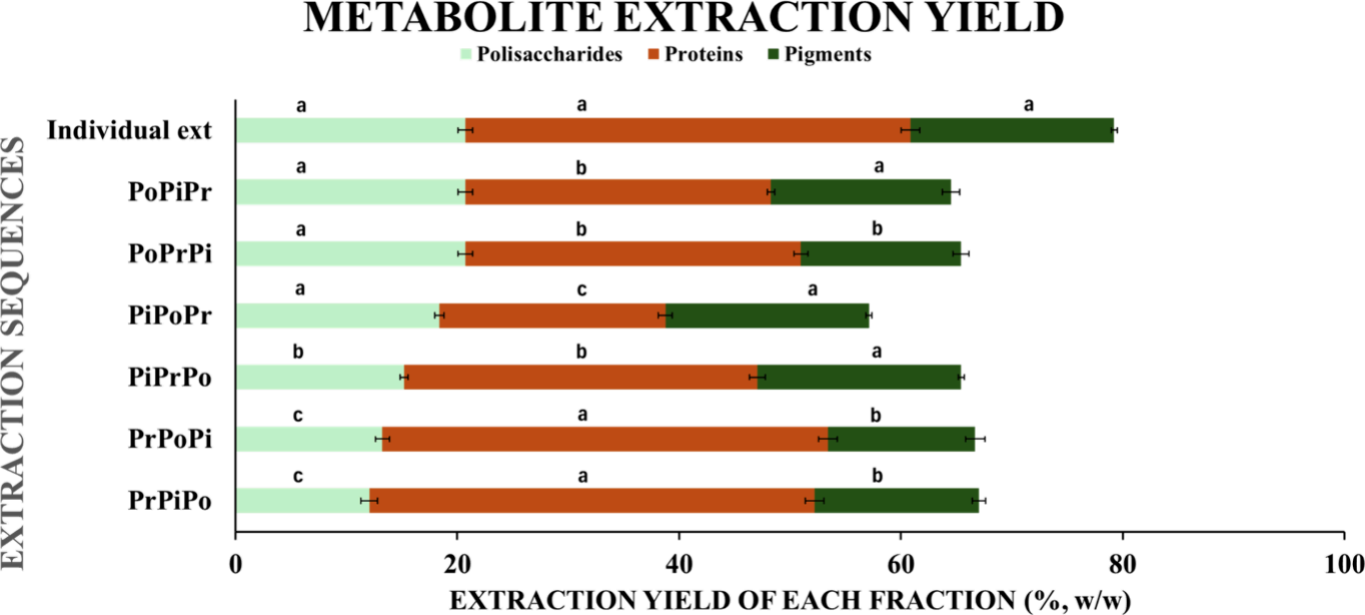
Effects of extraction sequence on the recovery yields of polysaccharides (Po), pigments (Pi), and proteins (Pr) from *C. vulgaris* . Values are presented as mean ± SD (*n* = 3 independent replicates). Normality of residuals and homogeneity of variances were assessed before analysis. Differences among groups were evaluated using one-way ANOVA, followed by Tukey’s HSD post hoc test (*p* < 0.05). Different letters indicate statistically significant differences (*p* < 0.05) within the same class of metabolites, comparing the respective yields across the tested sequences.

**Figure 7:**
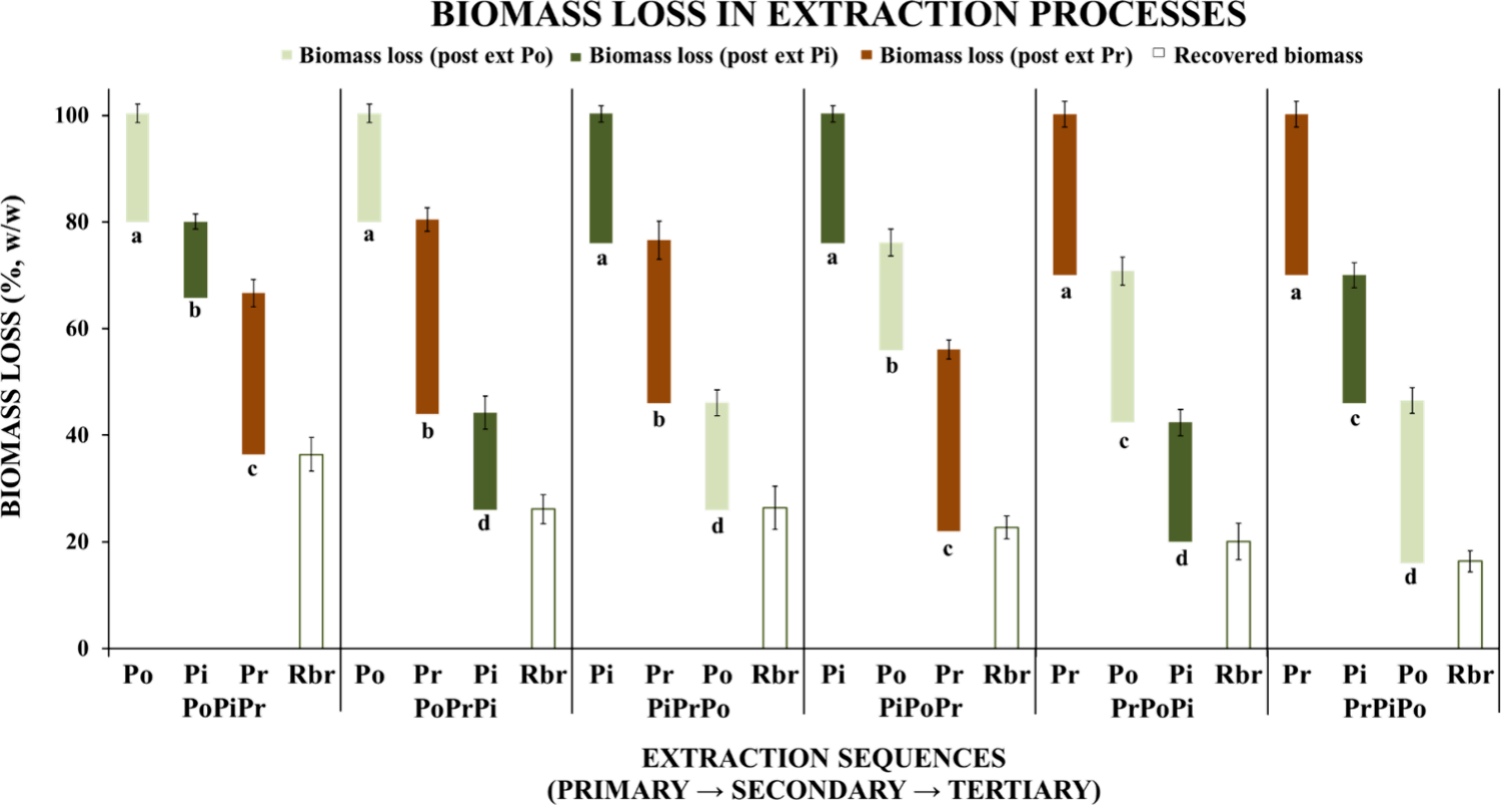
Effects of extraction sequence on biomass loss, expressed as percentage weight reduction (%, w/w). Values are presented as mean ± SD (*n* = 3 independent replicates). Normality of residuals and homogeneity of variances were assessed before analysis. Differences among groups were evaluated using one-way ANOVA followed by Tukey’s HSD post hoc test (*p* < 0.05). Different letters indicate statistically significant differences (*p* < 0.05) for the same class of metabolites extracted at different positions in the sequence (primary, secondary, and tertiary).

### 3.5 Effect of Sequential Extraction on Residual Biomass Recovery

Beyond metabolite yields, the extraction sequence strongly influences the quantity and quality of residual biomass, a key parameter for assessing overall biorefinery efficiency. We therefore evaluated how sequential processing affects biomass retention across the different stages of the cascade. Preservation of residual biomass ensures its availability for subsequent valorization pathways, including lipid recovery or agronomic applications.^4^

Results showed that sequential extraction of polysaccharides, pigments, and proteins from *C. vulgaris* resulted in a cumulative biomass loss at each step. As a consequence, secondary and tertiary extractions resulted in reduced yields.

As shown in Figure 7 and Supporting Information (Table S2), when polysaccharides were extracted as the first step (PoPiPr and PoPrPi), biomass retention remained high at approximately 80%. This corresponded to a biomass loss of only 20.35 ± 1.72%, indicating that hot-water extraction induced limited structural removal. In contrast, primary pigment extraction resulted in a slightly higher biomass loss (24.28 ± 1.5%), corresponding to a residual biomass of approximately 76%. This outcome is consistent with the moderate solubilizing capacity of ethanol. In contrast, primary protein extraction reduced biomass retention to approximately 70%, significantly lower than in polysaccharide-first extractions (ANOVA, *p* < 0.05). This effect is consistent with previous studies. Alkaline protein-solubilization steps promote extensive cell-wall disruption and solubilization of matrix components. These mechanisms contribute to accelerated biomass loss.^33,34^ After the second extraction step, the highest biomass retention (65.78 ± 1.82%) was observed in the PoPiPr sequence, whereas the lowest was recorded in PrPoPi (42.42 ± 2.32%).

Across all sequences, protein recovery steps accounted for the greatest biomass reductions (30–36%), regardless of their position. This finding confirms that alkaline extraction is inherently the most invasive treatment. In contrast, the impact of polysaccharide and pigment extraction was more strongly influenced by their position within the sequence. Notably, pigment extraction performed as a secondary step caused significantly less biomass reduction than polysaccharide extraction in the same position. When pigments were extracted in the final step, biomass loss reached its minimum, with values of 18.24 ± 3.06% for PoPrPi and 22.37 ± 2.48% for PrPoPi. These patterns suggest that proteins are more vulnerable to biomass loss from prior processing, possibly due to their progressive solubilization throughout the sequence. Overall, these results demonstrate that extraction order plays a decisive role in preserving biomass for downstream valorization. Among all tested sequences, PoPiPr retained the greatest amount of residual material after three steps (36.41 ± 2.52%), substantially more than sequences beginning with protein extraction (e.g., PrPiPo, 16.37 ± 1.78%). The residual biomass fraction remains suitable for additional recovery processes depending on the future intended use. The polarity-driven extraction logic can offer a balanced strategy for the biorefinery workflow.

### 3.6 Comparison between Extraction Yields and Residual Biomass Recovery

Identifying the most effective extraction strategy requires evaluating both key parameters, metabolite yields and the amount of biomass preserved after each step. A cross-analysis of these two parameters allows identification of extraction sequences that ensure efficient recovery of target fractions while preserving sufficient residual biomass for subsequent valorization steps. Because optimization of polysaccharide extraction represents a central objective of this research, further supported by the demonstrated biostimulant activity of the aqueous fraction on microgreens,^23^ the comparison focused on the PoPiPr and PoPrPi sequences. As shown in Figure 8, the PoPiPr sequence (polysaccharides → pigments → proteins) achieved yields of approximately 21% polysaccharides, 16% pigments, and 28% proteins, while retaining 36% of the initial biomass after the third extraction step. In contrast, the PoPrPi sequence (polysaccharides → proteins → pigments) produced similar polysaccharide yields (21%) and a slightly higher protein recovery (30%). However, pigment yield was lower (14%), and only 26% of the biomass was retained after processing. Although the two sequences exhibit similar total extraction yields, they differ markedly in their recovery profiles and in the amount of biomass available for downstream processing. The PoPiPr sequence enhances pigment recovery and preserves a larger biomass fraction. In contrast, PoPrPi slightly favors protein recovery but results in greater biomass loss. This reduction may negatively affect the overall efficiency and economic feasibility of a cascade biorefinery.

**Figure 8:**
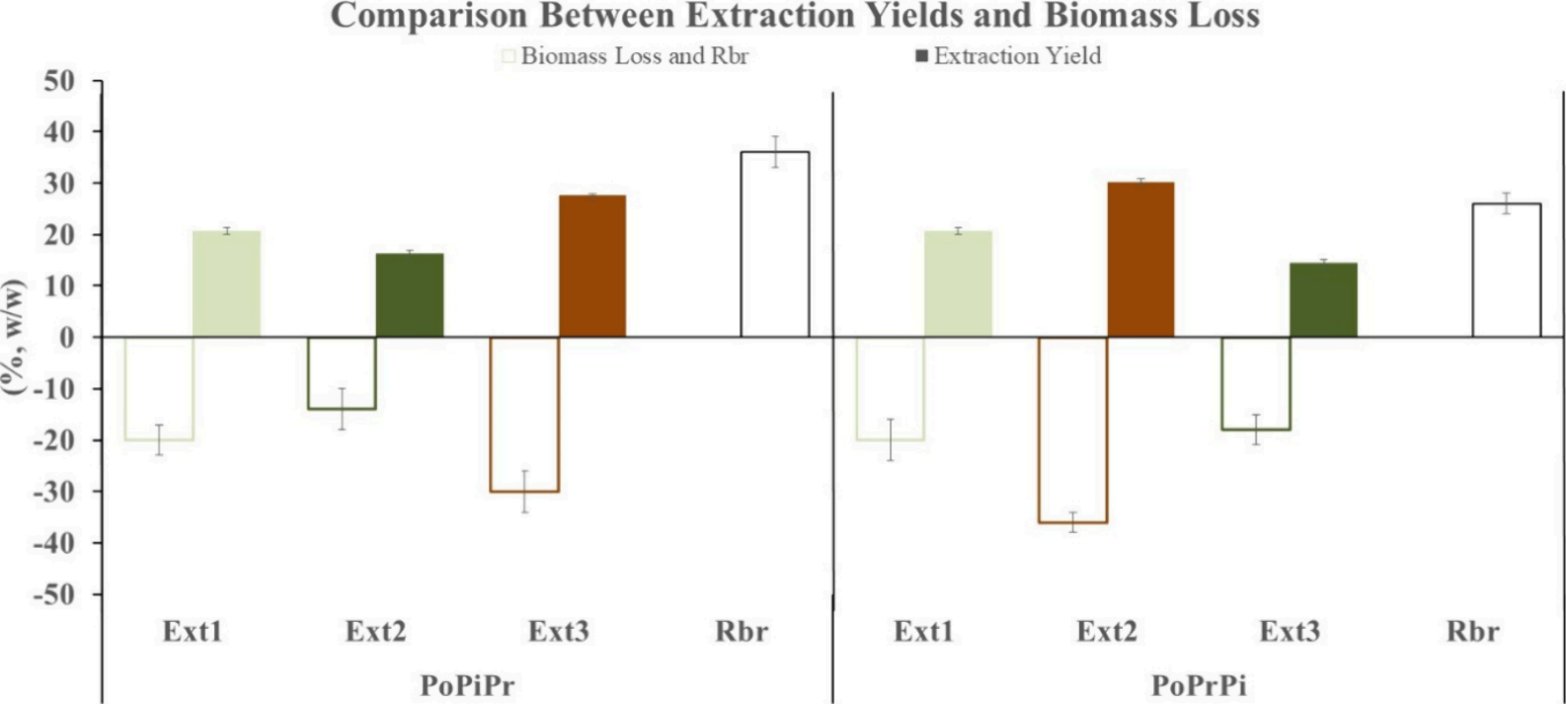
Comparative overview of biomass loss and extraction yields obtained from the two sequential extraction configurations (PoPiPr and PoPrPi). Negative values indicate biomass loss after each extraction step (Ext1–Ext3), whereas positive values represent metabolite yield and residual biomass recovery (Rbr) expressed as % w/w. Data are reported as mean ± SD. Statistical differences between configurations are detailed in Figures 6 and 7 [Fig fig7].

Overall, the PoPiPr sequence represents the most favorable compromise among the tested configurations. It enables balanced recovery of the three bioactive fractions while preserving a substantial portion of residual biomass. This retained biomass remains available for further valorization, including lipid extraction. These findings indicate that PoPiPr is the most advantageous extraction sequence within the evaluated cascade framework for integrated microalgal biorefinery applications.

### 3.7 Influence of Extraction Sequence on Reference Marker Content in Extracts

Beyond yield and biomass retention, the extraction sequence also shapes the biochemical profile of the recovered fractions. Thus, if and how different extraction orders affect the concentration of key reference markers in the extracts have been investigated. Variations in the content (mg g^–1^) of total neutral carbohydrates, proteins, and pigments demonstrate that extraction order significantly affects the biochemical composition of the recovered fractions. Regarding polysaccharides, aqueous extracts obtained either from untreated biomass or as the first step in the PoPiPr and PoPrPi sequences contained approximately 411.07 ± 7.79 mg g^–1^ of total neutral carbohydrates (Figure 9A). Sequences beginning with protein extraction (PrPoPi and PrPiPo) showed slightly higher polysaccharide concentrations, reaching 427.25 ± 6.32 and 436.48 ± 5.91 mg g^–1^, respectively. However, these differences were not statistically significant compared with the polysaccharide content measured in extracts recovered during the primary extraction step (*p* > 0.05). This trend likely reflects an apparent enrichment rather than a true increase in carbohydrate content. It may result from the removal of cell-wall-associated proteins during the alkaline extraction step. Such removal enhances the accessibility of residual polysaccharides to aqueous solvents.^4,33^ Conversely, pigment-first sequences (PiPoPr and PiPrPo) yielded lower polysaccharide contents (377.12 ± 6.23 and 371.64 ± 8.84 mg g^–1^), and both values were significantly lower than those obtained in the primary polysaccharide extraction (*p* < 0.05). This reduction may result from the partial solubilization or loss of loosely bound carbohydrate fractions during the ethanol-based pigment extraction step. Alcohol-rich environments are known to alter membrane permeability, remove low-molecular-weight saccharides, and induce cell-wall contraction. These effects can limit the subsequent release of water-soluble polysaccharides.^4,36,37^ Protein content also varies markedly depending on the extraction sequence. In untreated biomass and when proteins were extracted first (PrPiPo and PrPoPi), values were approximately 360.27 ± 4.36 mg g^–1^. When polysaccharides were extracted as the first step, protein levels were well preserved or slightly increased. In the PoPiPr sequence, protein content reached 392.53 ± 6.42 mg g^–1^, while in PoPrPi it increased to 407.32 ± 8.46 mg g^–1^. This preservation likely reflects the mild aqueous conditions, which do not promote significant protein denaturation (Figure 9B). In contrast, pigment-first sequences resulted in a significant reduction in protein content. In the PiPoPr sequence, protein levels decreased to 222.13 ± 7.76 mg g^–1^, and in PiPrPo to 231.54 ± 7.22 mg g^–1^ (*p* < 0.01). This trend is consistent with the known effects of ethanol-based solvents, which can disrupt membrane structures, extract low-molecular-weight proteins, and induce partial protein unfolding or dispersion.^4,22^ As expected, pigment preservation, assessed through lutein content, was highest in pigment-first sequences. Both PiPoPr and PiPrPo showed lutein levels of 1.09 ± 0.05 mg g^–1^ (Figure 9C), confirming that these extraction conditions best protect carotenoids. Lutein content decreased significantly when proteins were extracted first, reaching 0.77 ± 0.04 mg g^–1^ in PrPoPi and 0.79 ± 0.05 mg g^–1^ in PrPiPo. In polysaccharide-first sequences, lutein levels were moderately reduced to 0.82 ± 0.04 mg g^–1^ in PoPrPi and 0.88 ± 0.03 mg g^–1^ in PoPiPr. These reductions may be attributed to carotenoid degradation, oxidative stress, or partial solubilization under aqueous or alkaline conditions.^38,39^ No previous study has directly compared the three extraction orders evaluated here. However, the existing literature consistently indicates that carotenoid recovery from microalgae is highly sensitive to solvent polarity, pretreatments, and structural modifications of the residual biomass.^39^ Overall, extraction sequence shaped the biochemical composition of the resulting fractions in distinct ways. When polysaccharides were extracted first, proteins were preserved, although pigment levels were slightly reduced. Pigment-first sequences maximized pigment retention but led to pronounced protein loss. In contrast, protein-first sequences yielded slightly higher carbohydrate concentrations while causing the most substantial pigment degradation.

**Figure 9:**
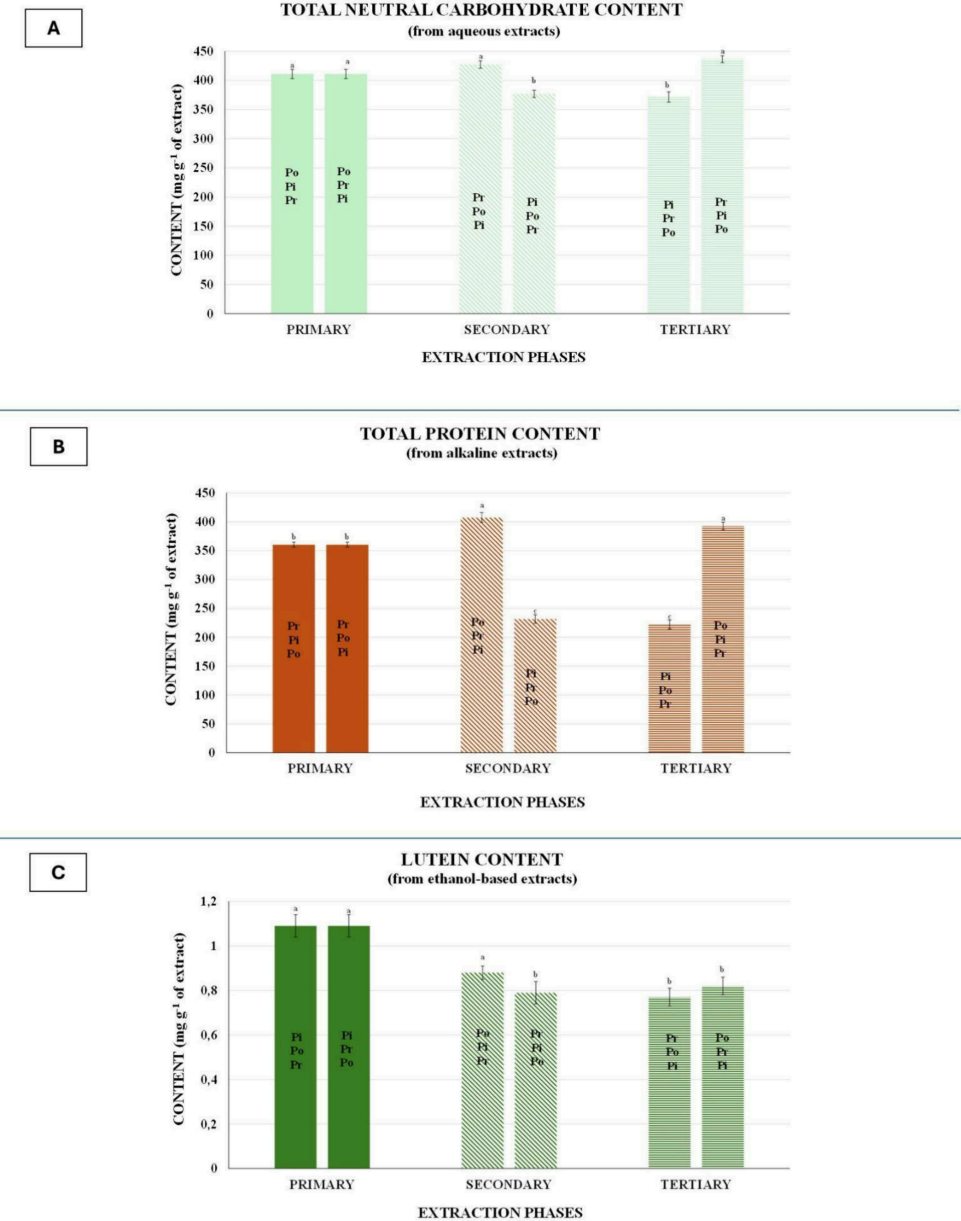
(A) Total neutral carbohydrate content in aqueous extracts, (B) total protein content in alkaline extracts, and (C) lutein content in ethanol-based extracts obtained across the developed extraction sequences. Data are expressed as mg of marker per g of extract (mean ± SD, *n* = 3). Normality of residuals and homogeneity of variances were assessed before analysis. Different letters denote statistically significant differences within each metabolite type, referring exclusively to comparisons of the same marker measured at different extraction steps of the sequences (Tukey’s HSD, *p* < 0.05).

Among all extraction strategies, PoPiPr emerged as the most balanced sequence in terms of biochemical preservation. As shown in Table 1, total neutral carbohydrate content remained essentially unchanged compared with untreated biomass (411.07 ± 7.79 mg g^–1^). Protein concentration was also well preserved at 392.53 ± 6.42 mg g^–1^, only slightly lower than the maximum observed in PoPrPi. Lutein content remained substantial at 0.88 ± 0.03 mg g^–1^. Importantly, this sequence also resulted in the highest residual biomass among the tested cascades (36%) (Table 1), thereby providing additional material for downstream valorization, including lipid extraction. Collectively, these results identify PoPiPr as the most favorable compromise between metabolite recovery, biochemical integrity, and biomass preservation within the evaluated framework.

**Table 1:**
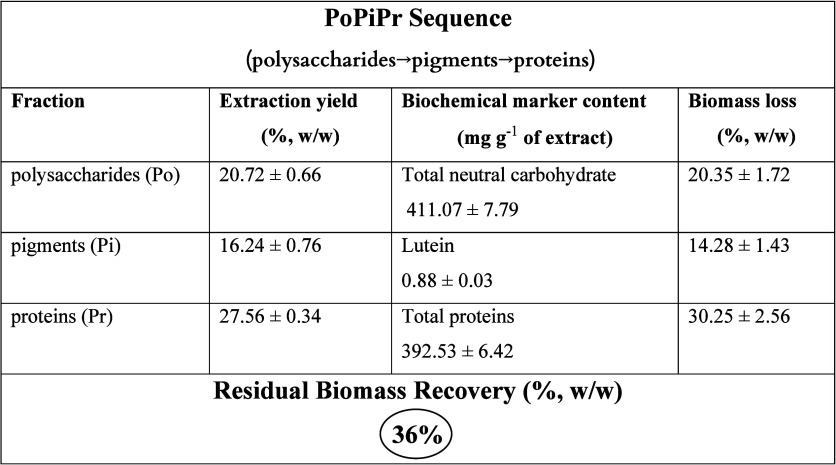
Summary of Extraction Yields, Marker Contents, and Biomass Loss for the PoPiPr Sequence

### 3.8 Evaluation of Phytotoxicity and Agronomic Performance of Residual Biomasses

To explore the potential agricultural reuse of extracted microalgal biomass, the phytotoxicity and biostimulant properties of residual fractions obtained from individual extraction processes were assessed. These evaluations provide insight into the suitability of sequentially processed biomasses as functional inputs for sustainable crop management. The residual *C. vulgaris* biomass obtained after the alkaline extraction step (CHL-RAlk) was not included in the agronomic assays. The material appeared highly degraded, sticky, and poorly soluble in water. These properties made it unsuitable for uniform dilution and foliar application. No signs of phytotoxicity were observed in any of the tested samples. The Germination Index (GI) significantly differed among treatments, indicating varying degrees of stimulatory activity. Among the biomasses, undiluted CHL-TQ (whole *C. vulgaris* biomass) produced the strongest stimulation on *Lactuca sativa* L. germination and root elongation, while its diluted forms (1:100 and 1:1000) maintained a consistent positive effect. CHL-RA (residual *C. vulgaris* biomass after aqueous extraction) exhibited the highest GI when undiluted, with comparable activity retained up to a 1:1000 dilution. CHL-RET (residual biomass after ethanol-based extraction), although less active at lower dilutions, showed the most pronounced biostimulant effect at the highest dilution (1:1000), suggesting a dose-dependent response. Based on these results, the most diluted concentration (1:1000) was selected for the greenhouse experiments. This concentration represented the lowest level tested that exhibited no phytotoxic effects while maintaining a consistent biostimulant response. This choice was further supported by its consistency with the dose–response behavior commonly reported for microalgal biostimulants. These products are typically effective at low application rates.^41^ Accordingly, the 1:1000 dilution represents a biologically relevant and agronomically realistic application level, comparable to those used for commercial *Chlorella*-based products.

Treatments included CHL-TQ 1:1000 (T3), CHL-RA 1:1000 (T4), and CHL-RET 1:1000 (T5), in comparison with two controls (T1, tap water) and a commercial biostimulant (T2, Agrialgae) (photographic comparison of theses being tested during the collection in Supporting Information, Figure S1). The commercial biostimulant Agrialgae was included as a functional reference rather than as a direct equivalence control. Both Agrialgae and the tested materials share a microalgal origin and a foliar mode of application. However, Agrialgae is a fully formulated and standardized commercial product. In contrast, the tested samples consist of whole or residual biomasses obtained through the cascade biorefinery process. Consequently, similarities in plant responses likely reflect the intrinsic bioactivity retained in the extracted biomasses. In contrast, observed differences may be attributed to formulation complexity, compositional standardization, and application rate. Although the chemical composition of the commercial biostimulant is proprietary, its application followed the recommended agronomic dose. This approach allowed a meaningful functional comparison under realistic cultivation conditions.

Physiological and agronomic parameters revealed significant treatment-dependent variations (Table 2). Stomatal conductance was the highest in T2, significantly exceeding T1, T3, T4, and T5, which exhibited comparable values. Soil water content followed a similar trend, being greatest in T2 and T3, suggesting that CHL-TQ improved leaf gas exchange and water-use efficiency. Although plant height did not differ significantly among treatments, leaf number was the highest in T5, followed by T3, indicating that CHL-RET and CHL-TQ promoted vegetative proliferation. This finding is consistent with previous studies reporting that microalgal extracts enhance leaf formation by stimulating photosynthetic activity and cytokinin-like responses in lettuce and tomato plants.^42,43^ Aboveground fresh weight was significantly greater in T5 and T3 compared with the control, while belowground fresh weight peaked in T4, suggesting a differentiated biomass allocation pattern depending on the extract applied. The enhanced root development observed in CHL-RA-treated plants (residual biomass after aqueous extraction) is likely attributable to residual nitrogenous compounds, amino acids, and mineral nutrients. These components remained unextracted during the hot-water process. Such molecules are known to exert auxin-like and signaling effects that stimulate root elongation and branching, improving nutrient uptake and overall root vigor.^43^ Similar results were reported by Ammaturo et al.^44^ and La Bella et al.,^3^ who found enhanced root growth in lettuce following treatments with *C. vulgaris* extracts or their cell-wall residues. This finding suggests that, even after the extraction of polar metabolites, the remaining biomass retains sufficient biochemical complexity. Such complexity may contribute to enhanced belowground biomass development and improved resource-use efficiency. Crop Water Productivity (CWP) and Nitrogen Agronomic Efficiency (NAE) were also the highest in T5, followed by T3, confirming the strong biostimulant potential of the residual after ethanol-based extraction (CHL-RET) and whole (CHL-TQ) biomasses. These results are consistent with previous studies showing that microalgal residues and extracts can improve plant productivity. Reported effects include modulation of physiological responses, enhanced nutrient assimilation, and increased water-use efficiency under controlled conditions.^17,45^ A comparative photographic overview of lettuce plants under the five treatments is provided in the Supporting Information (Figure S1) to visually support the agronomic trends described.

**Table 2:** Physiological and Agronomic Parameters with Statistically Significant Differences Among Treatments^a^

**treatments**	**T1**		**T2**		**T3**		**T4**		**T5**	
stomatal conductance (mmol H_2_O m^–2^ s^–1^)	267.00	b	375.00	a	204.30	b	227.00	b	258.00	b
SPAD	17.65	a	14.20	a	14.83	a	14.37	a	18.67	a
plant height (cm)	20.75	a	21.00	a	19.33	a	21.00	a	20.33	a
soil water content (%)	14.95	c	33.65	a	26.40	b	16.07	c	15.20	c
number of leaves (no. pot^–1^)	31.50	bc	29.00	c	38.50	ab	29.50	c	43.00	a
aboveground fresh weight (g pot^–1^)	23.36	c	17.11	c	32.31	ab	25.70	bc	34.38	a
belowground fresh weight (g pot^–1^)	32.75	b	42.94	ab	37.02	ab	52.56	a	43.94	a
CWP (g mL^–1^ pot^–1^)	0.021	bc	0.019	c	0.026	ab	0.020	c	0.029	a
NAE (g g^–1^ pot^–1^)	0.004	bc	0.004	c	0.005	ab	0.004	c	0.005	a

a T1: tap water; T2: commercial biostimulant; T3: CHL-TQ 1:1000; T4: CHL-RA 1:1000; T5: CHL-RET 1:1000; SPAD: chlorophyll index; TDR = time-domain reflectometry; CWP: crop water productivity; NAE: nitrogen agronomic efficiency. Different letters within each row indicate significant differences according to Tukey’s HSD (*p* < 0.05).

Some treatment effects were moderate in magnitude. However, their biological relevance is supported by the consistent responses observed across multiple independent agronomic and physiological parameters. These parameters include biomass accumulation, leaf number, crop water productivity, and nitrogen agronomic efficiency. Moreover, the experiments were conducted under controlled greenhouse conditions with limited environmental stress. Under such conditions, biostimulant-induced effects are often attenuated. The detection of statistically significant differences under these conservative conditions therefore supports the robustness and functional relevance of the observed responses. Overall, the residual biomasses recovered from the sequential extraction processes exhibited consistent biostimulant activity and performed comparably to the commercial reference product across several parameters. These results support the view that microalgal residues, particularly those derived from early extraction stages, can be repurposed as sustainable agricultural inputs within circular biorefinery frameworks.

### 3.9 Process Scalability and Sustainability: Limitations and Outlook

From a process engineering perspective, scalability and sustainability of cascade biorefineries depend not only on product yields but also on solvent use, energy demand, and operational complexity. In the present study, sustainability considerations derive from solvent-efficient extraction steps, the avoidance of high-pressure or supercritical systems, and the use of unit operations already established at industrial scale.^4,22^ Mechanical disruption and spray drying are the most energy-demanding stages. However, both are widely implemented in industrial microalgal processing and supported by consolidated scale-up strategies.^9,15^ Compared with lipid-first schemes requiring solvent-intensive extraction or supercritical CO_2_ systems, the polarity-driven cascade prioritizes aqueous and ethanol-based steps, thereby simplifying solvent recovery and reducing environmental and safety burdens.^7,8^ Alkaline protein extraction, although chemically intensive, is performed under moderate conditions and reflects conventional industrial practice for protein solubilization.^33^ Operational sustainability further depends on solvent and water management. At the cultivation stage, a biomass concentration of 4 g L^–1^ implies a substantial associated water volume. However, industrial microalgal production systems routinely implement medium recycling after nutrient rebalancing, significantly reducing net freshwater demand. Closed-loop systems commonly achieve high reuse efficiencies, thereby mitigating the overall water footprint of biomass production. Both water and ethanol can be efficiently recovered through distillation or membrane-based separation, while alkaline streams can be neutralized and partially recycled, reducing effluent generation. At larger scale, increasing solid loading or implementing counter-current extraction configurations would further decrease solvent-to-biomass ratios. Such process intensification strategies are expected to improve overall operational efficiency compared with laboratory-scale conditions. Importantly, extraction sequencing directly affects material efficiency and downstream integration potential.^7–9,40^ Sequences associated with greater cumulative biomass loss inherently require larger solvent volumes and generate increased residual streams. In this respect, configurations preserving higher residual biomass fractions, such as Po → Pi → Pr, are expected to minimize overall solvent consumption and process waste generation. This strategy aligns with multiproduct biorefinery principles aimed at improving economic resilience and resource efficiency.^10,11^ The present study demonstrates that residual biomasses retain functional properties suitable for further valorization. However, a comprehensive quantitative compositional analysis of these residues was not conducted. Detailed analysis of residual macromolecular fractions, ash content, and elemental composition would strengthen correlations between residue chemistry and specific downstream applications. Future investigations should therefore integrate systematic compositional profiling with techno-economic and energy assessments to quantitatively substantiate scalability and guide industrial implementation pathways.

## 4 Conclusions

This study presents a validated sequential biorefinery model for *C. vulgaris* . The model integrates the recovery of polysaccharides, pigments, and proteins from high-quality biomass cultivated in a controlled indoor system. Among the six extraction schemes tested, the PoPiPr sequence (polysaccharides → pigments → proteins) proved to be the most effective. It provided high metabolite yields, preserved biochemical integrity, and retained 36% residual biomass for further valorization. The results demonstrate that extraction order critically influences both recovery efficiency and extract quality, and that a polarity-driven sequence enhances overall process performance. The PoPiPr configuration was designed to minimize solvent consumption and preserve thermolabile compounds. This design makes it a promising candidate for future scale-up investigations and environmentally conscious processing strategies. Beyond metabolite recovery, biomass residues generated after each extraction sstep retained biologically active properties. These findings indicate that single-stage residues preserve functional integrity and remain suitable for secondary valorization pathways. Their demonstrated biostimulant activity on *Lactuca sativa* L. confirms that microalgal residues, rich in amino acids, minerals, and signaling compounds, can be reused as agronomic inputs. This reuse contributes to nutrient recycling within an integrated biomass utilization framework.

Greenhouse trials showed performance comparable to that of a commercial biostimulant. These results highlight the dual role of microalgal residual biomass as both a source of bioactive compounds and a potential sustainable agricultural input under controlled experimental conditions. Such dual functionality strengthens the link between biotechnology and sustainable crop production, paving the way for integrated biorefinery models in which multiple biomass fractions contribute measurable value. Compared with biodiesel-first pathways, the cascade biorefinery model may offer advantages in terms of product diversification and resource efficiency. However, dedicated techno-economic and life cycle assessments are required to quantitatively validate its economic and environmental performance. Future work should focus on scaling the process and expanding its application to other microalgal species and crop systems. Such efforts would support the transition toward more resilient, low-input agricultural models.

## Supplementary Material



## Notes

Additional information on extraction yields (%, w/w) of polysaccharides (Po), proteins (Pr), and pigments (Pi) in each sequence according to their extraction position (Table S1); biomass loss (%, w/w) during each extraction step across sequential extraction schemes (Table S2); and photographic comparison of theses being tested during the collection (Figure S1) (PDF)

## †

Department of Chemistry and Biology, University of Salerno, 84084 Fisciano, SA, Italy

## Abbreviations

Po: polysaccharides
Pi: pigments
Pr: proteins
PoPiPr: sequential extraction of polysaccharides, pigments, and proteins
PoPrPi: sequential extraction of polysaccharides, proteins, and pigments
PiPoPr: sequential extraction of pigments, polysaccharides, and proteins
PiPrPo: sequential extraction of pigments, proteins, and polysaccharides
PrPoPi: sequential extraction of proteins, polysaccharides, and pigments
PrPiPo: sequential extraction of proteins, pigments, and polysaccharides
UV–vis: ultraviolet–visible
*C. vulgaris*: *Chlorella vulgaris*
EROI: Energy Return on Investiment
CHL-A: polysaccharide-rich extract (from *Chlorella vulgaris* biomass)
SEM: scanning electron microscopy
EtOH: ethanol
BSA: bovine serum albumin
CHL-P: pigment-rich extract (from *Chlorella vulgaris* biomass)
CHL-Alk: Alkaline extract from *Chlorella vulgaris* biomass
GI: germination index
CHL-TQ: whole *Chlorella vulgaris* biomass
CHL-RA: residual *Chlorella vulgaris* biomass after aqueous extraction
CHL-RET: residual *Chlorella vulgaris* biomass after ethanol-based extraction
CHL-RAlk: residual *Chlorella vulgaris* biomass after alkaline extraction
CWP: crop water productivity
NAE: nitrogen agronomic efficiency
DW: dried weight
Rbr: residual biomass recovery

## References

[1] Eilam, Y. ; Khattib, H. ; Pintel, N. ; Avni, D. Microalgae—Sustainable Source for Alternative Proteins and Functional Ingredients Promoting Gut and Liver Health. Global Challenges; John Wiley and Sons Inc, 2023.10.1002/gch2.202200177PMC1019062037205927

[2] Kuo C. M., Yang Y. C., Zhang W. X., Wu J. X., Chen Y. T., Lin C. H., Lin M. W., Lin C. S. (2023). A Low-Cost Fertilizer
Medium Supplemented with Urea for the Lutein Production of Chlorella
Sp. and the Ability of the Lutein to Protect Cells against Blue Light
Irradiation. Bioengineering.

[3] La
Bella E., Baglieri A., Rovetto E. I., Stevanato P., Puglisi I. (2021). Foliar Spray Application of Chlorella Vulgaris Extract:
Effect on the Growth of Lettuce Seedlings. Agronomy.

[4] Safi C., Zebib B., Merah O., Pontalier P. Y., Vaca-Garcia C. (2014). Morphology, Composition, Production,
Processing and
Applications of Chlorella Vulgaris: A Review. Renewable and Sustainable Energy Reviews.

[5] Vasistha S., Khanra A., Clifford M., Rai M. P. (2021). Current Advances
in Microalgae Harvesting and Lipid Extraction Processes for Improved
Biodiesel Production: A Review. Renewable and
Sustainable Energy Reviews.

[6] Khanra A., Vasistha S., Rai M. P., Cheah W. Y., Khoo K. S., Chew K. W., Chuah L. F., Show P. L. (2022). Green Bioprocessing
and Applications of Microalgae-Derived Biopolymers as a Renewable
Feedstock: Circular Bioeconomy Approach. Environ.
Technol. Innov..

[7] Wijffels R. H., Barbosa M. J., Eppink M. H. M. (2010). Microalgae for
the Production of
Bulk Chemicals and Biofuels. Biofuels Bioprod.
Biorefin..

[8] Olguín E. J., Sánchez-Galván G., Arias-Olguín I. I., Melo F. J., González-Portela R. E., Cruz L., De Philippis R., Adessi A. (2022). Microalgae-Based Biorefineries:
Challenges
and Future Trends to Produce Carbohydrate Enriched Biomass, High-Added
Value Products and Bioactive Compounds. Biology.

[9] Slegers P. M., Olivieri G., Breitmayer E., Sijtsma L., Eppink M. H. M., Wijffels R. H., Reith J. H. (2020). Design
of Value Chains for Microalgal
Biorefinery at Industrial Scale: Process Integration and Techno-Economic
Analysis. Front. Bioeng. Biotechnol..

[10] Prabha S., Vijay A. K., Paul R. R., George B. (2022). Cyanobacterial Biorefinery:
Towards Economic Feasibility through the Maximum Valorization of Biomass. Sci. Total Environ..

[11] Siddiki S. Y. A., Mofijur M., Kumar P. S., Ahmed S. F., Inayat A., Kusumo F., Badruddin I. A., Khan T. M. Y., Nghiem L. D., Ong H. C., Mahlia T. M. I. (2022). Microalgae
Biomass as a Sustainable
Source for Biofuel, Biochemical and Biobased Value-Added Products:
An Integrated Biorefinery Concept. Fuel.

[12] Mendes A. R., Spínola M. P., Lordelo M., Prates J. A. M. (2024). Chemical Compounds,
Bioactivities, and Applications of Chlorella Vulgaris in Food, Feed
and Medicine. Applied Sciences (Switzerland).

[13] Assunção J., Amaro H. M., Tavares T., Malcata F. X., Guedes A. C. (2023). Effects
of Temperature, PH, and NaCl Concentration on Biomass and Bioactive
Compound Production by Synechocystis Salina. Life.

[14] Karabulut G., Purkiewicz A., Goksen G. (2024). Recent Developments and Challenges
in Algal Protein and Peptide Extraction Strategies, Functional and
Technological Properties, Bioaccessibility, and Commercial Applications. Comprehensive Reviews in Food Science and Food Safety.

[15] Becker E. W. (2007). Micro-Algae
as a Source of Protein. Biotechnology Advances..

[16] Rizwan M., Mujtaba G., Memon S. A., Lee K., Rashid N. (2018). Exploring
the Potential of Microalgae for New Biotechnology Applications and
beyond: A Review. Renewable and Sustainable
Energy Reviews.

[17] Di
Serio A., Aquino G., Del Prete F., Sansone F., Salviati E., Basilicata M. G., Manfra M., Campiglia P., Ronga D., Pepe G. (2025). Protein Hydrolysates
Derived from Residual after Polysaccharide Extraction of Chlorella
Vulgaris Biomass Improves Yield and Quality of Baby Leaf Lettuce. Sci. Rep..

[18] Puglisi I., La Bella E., Rovetto E. I., Lo Piero A. R., Baglieri A. (2020). Biostimulant
Effect and Biochemical Response in Lettuce Seedlings Treated with
a Scenedesmus Quadricauda Extract. Plants.

[19] Ergun O., Dasgan H. Y., Isik O. (2020). Effects of
Microalgae Chlorella Vulgaris
on Hydroponically Grown Lettuce. Acta Hortic..

[20] Ansari F. A., Shriwastav A., Gupta S. K., Rawat I., Bux F. (2017). Exploration
of Microalgae Biorefinery by Optimizing Sequential Extraction of Major
Metabolites from Scenedesmus Obliquus. Ind.
Eng. Chem. Res..

[21] Suganya T., Nagendra Gandhi N., Renganathan S. (2013). Production of Algal Biodiesel from
Marine Macroalgae Enteromorpha Compressa by Two Step Process: Optimization
and Kinetic Study. Bioresour. Technol..

[22] Bleakley S., Hayes M. (2017). Algal Proteins: Extraction,
Application, and Challenges Concerning
Production. Foods.

[23] Del
Prete F., Esposito T., Pane C., Manganiello G., Pepe G., Salviati E., Campiglia P., Mencherini T., Sansone F., Aquino R. P. (2025). Extract from Chlorella
Vulgaris: Production, Characterization, and Effects on the Germination,
Growth and Metabolite Profile of Eruca Sativa Microgreens. Ind. Crops Prod..

[24] Rippka E., Deruelles J., Waterbury N. B. (1979). Generic
Assignments, Strain Histories
and Properties of Pure Cultures of Cyanobacteria. Microbiology.

[25] Sansone F., Esposito T., Mencherini T., Del Prete F., Cannoniere A. L., Aquino R. P. (2023). Exploring Microencapsulation
Potential:
Multicomponent Spray Dried Delivery Systems for Improvement of Chlorella
Vulgaris Extract Preservation and Solubility. Powder Technol..

[26] Pierce B. R. (1977). Wrongful
Death Action May Not Be Maintained Under Strict Liability Statute. Mercer L. Rev..

[27] Zucconi F. (1981). Evaluating Toxicity
of Immature Compost. Biocycle.

[28] Ronga D., Biazzi E., Parati K., Carminati D., Carminati E., Tava A. (2019). Microalgal Biostimulants
and Biofertilisers
in Crop Productions. Agronomy.

[29] Lin J., Deng J., Huang Z., Dong H., Chang A., Zhu H. (2023). Physicochemical and
Structural Characterization of Alkali-Treated
Biopolymer Sphingan WL Gum from Marine Sphingomonas Sp. WG. ACS Omega.

[30] Chaiklahan R., Chirasuwan N., Triratana P., Loha V., Tia S., Bunnag B. (2013). Polysaccharide
Extraction from Spirulina Sp. and Its
Antioxidant Capacity. Int. J. Biol. Macromol..

[31] Mendes R. L., Reis A. D., Palavra A. F. (2006). Supercritical
CO2 Extraction of γ-Linolenic
Acid and Other Lipids from Arthrospira (Spirulina)­Maxima: Comparison
with Organic Solvent Extraction. Food Chem..

[32] Chemat F., Rombaut N., Sicaire A. G., Meullemiestre A., Fabiano-Tixier A. S., Abert-Vian M. (2017). Ultrasound Assisted Extraction of
Food and Natural Products. Mechanisms, Techniques, Combinations, Protocols
and Applications. A Review. Ultrasonics Sonochemistry.

[33] Ursu A. V., Marcati A., Sayd T., Sante-Lhoutellier V., Djelveh G., Michaud P. (2014). Extraction, Fractionation
and Functional
Properties of Proteins from the Microalgae Chlorella Vulgaris. Bioresour. Technol..

[34] Benelhadj S., Gharsallaoui A., Degraeve P., Attia H., Ghorbel D. (2016). Effect of
PH on the Functional Properties of Arthrospira (Spirulina) Platensis
Protein Isolate. Food Chem..

[35] Ferreira A. S., Ferreira S. S., Correia A., Vilanova M., Silva T. H., Coimbra M. A., Nunes C. (2020). Reserve, Structural
and Extracellular
Polysaccharides of Chlorella Vulgaris: A Holistic Approach. Algal Res..

[36] Templeton D. W., Laurens L. M. L. (2015). Nitrogen-to-Protein
Conversion Factors Revisited for
Applications of Microalgal Biomass Conversion to Food. Feed and Fuel. Algal Res..

[37] Matos Â. P., Feller R., Moecke E. H. S., de Oliveira J. V., Junior A. F., Derner R. B., Sant’Anna E. S. (2016). Chemical
Characterization of Six Microalgae with Potential Utility for Food
Application. J. Am. Oil Chem. Soc..

[38] Montuori E., Lima S., Marchese A., Scargiali F., Lauritano C. (2024). Lutein Production and Extraction
from Microalgae: Recent
Insights and Bioactive Potential. International
Journal of Molecular Sciences.

[39] Pasquet V., Chérouvrier J. R., Farhat F., Thiéry V., Piot J. M., Bérard J. B., Kaas R., Serive B., Patrice T., Cadoret J. P., Picot L. (2011). Study on the Microalgal
Pigments Extraction Process: Performance of Microwave Assisted Extraction. Process Biochemistry.

[40] Banerjee S., Ray A., Das D. (2021). Optimization
of Chlamydomonas Reinhardtii Cultivation
with Simultaneous CO2 Sequestration and Biofuels Production in a Biorefinery
Framework. Sci. Total Environ..

[41] Prisa D., Spagnuolo D. (2023). Plant Production
with Microalgal Biostimulants. Horticulturae.

[42] Khan M. I., Shin J. H., Kim J. D. (2018). The Promising
Future of Microalgae:
Current Status, Challenges, and Optimization of a Sustainable and
Renewable Industry for Biofuels, Feed, and Other Products. Microbial Cell Factories.

[43] Colla G., Rouphael Y. (2020). Microalgae: New Source
of Plant Biostimulants. Agronomy.

[44] Ammaturo C., Pacheco D., Cotas J., Formisano L., Ciriello M., Pereira L., Bahcevandziev K. (2023). Use of Chlorella
Vulgaris and Ulva Lactuca as Biostimulant on Lettuce. Appl. Sci..

[45] Rouphael Y., Colla G. (2020). Editorial: Biostimulants in Agriculture. Frontiers
in Plant Science.

