# Supplementary material for: Cascade Biorefinery of Chlorella vulgaris: Optimized Extraction Sequencing for Sustainable Whole-Biomass Valorization
Source: ACS Sustain Chem Eng. 2026 Mar 25;14(13):6455–70. doi: 10.1021/acssuschemeng.5c13850 (PMC13073689; doi:10.1021/acssuschemeng.5c13850)
Supplement: Supplementary file 1 [file sc5c13850_si_001.pdf]

Supporting Information

Cascade biorefinery of *Chlorella vulgaris*: optimized extraction sequencing for sustainable whole-biomass valorization.

Francesco Del Prete[§]*, *Francesca Sansone*[§], *Francesca Fortunato*[§], *Tiziana Esposito*[§], *Teresa Mencherini*[§], *Annamaria Di Serio*[†§], *Domenico Ronga*[§], *Rita. P. Aquino*[§]

* Corresponding author: Francesco Del Prete E-mail: fdelprete@unisa.it

§ Department of Pharmacy, University of Salerno, 84084 Fisciano, SA, Italy

†Department of Chemistry and Biology, University of Salerno, 84084 Fisciano, SA, Italy

This Supporting Information contains:

3 pages

2 supplementary tables

1 supplementary figure

Table S1: Extraction yields (%, w/w) of polysaccharides (Po), proteins (Pr), and pigments (Pi) quantified in each sequence according to their extraction position (first, second, or third step). Data are reported as mean ± SD (n = 3).

Extraction Sequence	Polysaccharides Po (%)	Proteins Pr (%)	Pigments Pi (%)	Total Recovered
**PoPiPr**	20.72±0.66	27.56±0.34	16.24±0.76	64.52%
**PoPrPi**	20.72±0.66	30.27±0.65	14.42±0.73	65.41%
**PiPrPo**	18.38±0.42	20.38±0.64	18.36±0.27	57.12%
**PiPoPr**	15.23±0.36	31.85±0.72	18.36±0.27	65.44%
**PrPoPi**	13.25±0.65	40.16±0.83	13.28±0.87	66.69%
**PrPiPo**	12.06±0.76	40.16±0.83	14.82±0.59	67.04%

Table S2: Biomass loss (%. w/w) during each extraction step across sequential extraction schemes. Values represent mean ± SD (n = 3). Biomass loss is expressed as a percentage of dry weight reduction after each step. Total biomass loss was calculated cumulatively across the three phases.

Extraction Sequence	Step 1 Loss (%)	Step 2 Loss (%)	Step 3 Loss (%)	Total biomass loss (%)	Residual biomass recovery
**PoPiPr**	20.35±1.72	14.28±1.43	30.25±2.56	64.42±3.60	36.41±3.15
**PoPrPi**	20.35±1.72	36.44±2.24	18.24±3.06	75.24±3.08	26.12±2.76
**PiPrPo**	24.28±1.50	30.56±3.60	20.08±2.45	74.65±2.46	26.38±4.07
**PiPoPr**	24.28±1.50	20.12±2.52	34.03±1.78	78.23±1.96	22.67±2.16
**PrPoPi**	30.18±2.43	28.32±2.63	22.37±2.48	80.39±2.47	20.06±3.4
**PrPiPo**	30.18±2.43	24.02±2.38	30.51±2.44	84.33±3.04	16.37±2.00

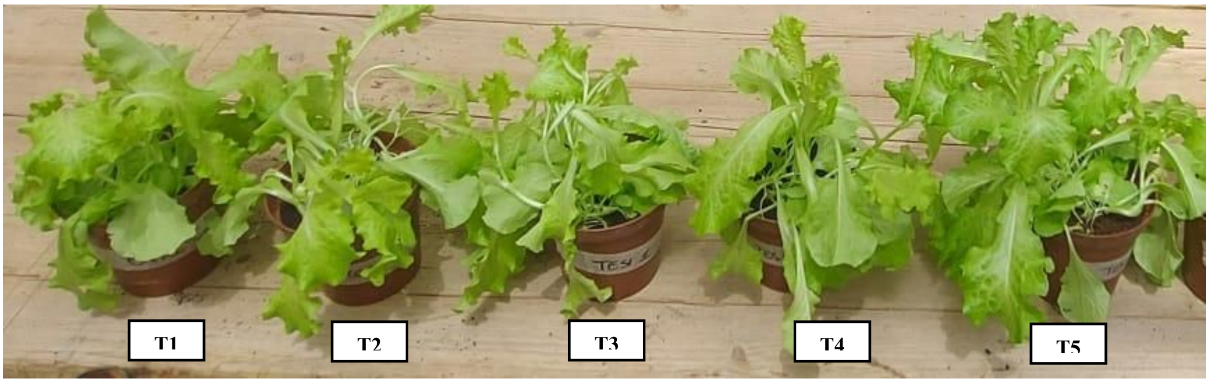

Figure S1: Photographic comparison of theses being tested during the collection: T1: control; T2: commercial biostimulant; T3: CHL-TQ 1:1000; T4: CHL-RA 1:1000; T5: CHL-RET 1:1000
